# Collective Efficacy: Development and Validation of a Measurement Scale for Use in Public Health and Development Programmes

**DOI:** 10.3390/ijerph15102139

**Published:** 2018-09-28

**Authors:** Maryann G. Delea, Gloria D. Sclar, Mulat Woreta, Regine Haardörfer, Corey L. Nagel, Bethany A. Caruso, Robert Dreibelbis, Abebe G. Gobezayehu, Thomas F. Clasen, Matthew C. Freeman

**Affiliations:** 1 Department of Disease Control, Faculty of Infectious & Tropical Diseases, London School of Hygiene & Tropical Medicine, London WC1E 7HT, UK; robert.dreibelbis@lshtm.ac.uk (R.D.); thomas.f.clasen@emory.edu (T.F.C.); 2 Department of Environmental Health, Rollins School of Public Health, Emory University, Atlanta, GA 30322, USA; gloria.sclar@emory.edu (G.D.S.); bethany.caruso@emory.edu (B.A.C.); matthew.freeman@emory.edu (M.C.F.); 3 Emory Ethiopia, Bahir Dar, Addis Ababa, Ethiopia; mworeta@manhep.org (M.W.); abebe.g.gobezayehu@emory.edu (A.G.G.); 4 Department of Behavioral Sciences and Health Education, Rollins School of Public Health, Emory University, Atlanta, GA 30322, USA; regine.haardoerfer@emory.edu; 5 College of Nursing, University of Arkansas for Medical Sciences, Little Rock, AR 72205, USA; cnagel@uams.edu

**Keywords:** collective efficacy, WASH, behaviour change, gender, behavioural control, collective action, cooperative behaviour, community-based interventions, factor analysis

## Abstract

Impact evaluations of water, sanitation, and hygiene interventions have demonstrated lower than expected health gains, in some cases due to low uptake and sustained adoption of interventions at a community level. These findings represent common challenges for public health and development programmes relying on collective action. One possible explanation may be low collective efficacy (CE)—perceptions regarding a group’s ability to execute actions related to a common goal. The purpose of this study was to develop and validate a metric to assess factors related to CE. We conducted this research within a cluster-randomised sanitation and hygiene trial in Amhara, Ethiopia. Exploratory and confirmatory factor analyses were carried out to examine underlying structures of CE for men and women in rural Ethiopia. We produced three CE scales: one each for men and women that allow for examinations of gender-specific mechanisms through which CE operates, and one 26-item CE scale that can be used across genders. All scales demonstrated high construct validity. CE factor scores were significantly higher for men than women, even among household-level male-female dyads. These CE scales will allow implementers to better design and target community-level interventions, and examine the role of CE in the effectiveness of community-based programming.

## 1. Introduction

It has become commonplace in international development to intervene in communities with interventions that require collective action without first gauging the communities’ perceptions regarding their ability and autonomy to engender and maintain change. Such is the case with many water, sanitation, and hygiene (WASH) interventions, some of which require collective action before first assessing whether reliance on shared agency is a realistic expectation, others of which neglect to address important factors of collective behaviour. This, perhaps, may be an artefact of common programme approaches that tend to address independent, individual and household-level behaviours while aiming for change at higher levels, such as villages, communities, or other collectives of people. Yet, in order to facilitate interdependent adoption of improved collective behaviours, evidence suggests it is important for interventions to address underlying factors that facilitate action and change at those levels [1,2,3,4]. Overlooking or underestimating the role of collective behavioural factors, such as behavioural control perceptions (e.g., agency-related factors such as self- and collective efficacy) and social schemas (e.g., social norms) in the uptake of community-based interventions may, in turn, attenuate intervention impact [2,4].

Evidence suggests that collective action is required for WASH interventions to reach the coverage and use levels likely required to realise health gains through “herd protection” [5,6,7]. Results from rigorously designed and evaluated WASH studies demonstrate lower than expected impact of WASH interventions on health [8,9,10], in some cases due to poor intervention uptake and sustained adoption. When interpreting these findings, it is important to consider the implementation approaches and intervention techniques that were employed, as well as the level at which these interventions were targeted (e.g., individual, household, group, community). It is pertinent to question whether the WASH sector is considering potentially important behavioural antecedents (i.e., upstream behavioural factors predictive of downstream behavioural, health, and development impacts) in their theories of change, intervention designs, and programme evaluations.

In many sectors, including but not limited to WASH, community-based programmes that target higher order groups (e.g., households, villages, health centres, government ministries) often inadequately address factors of collective behaviour in their intervention design and implementation strategies [11]. Collective efficacy (CE) is one such factor. CE is a latent social construct that encompasses a combination of cognitive and socio-structural factors which facilitate peoples’ shared beliefs in their collective power, or ability to come together to execute actions related to a common goal [12,13]. As with other social constructs, CE is complex, and draws on multiple sub-construct domains, such as social cohesion, social control, and cognitive and structural social capital. In addition to shaping a group’s decision to pursue common goals, collective efficacy also influences the amount of effort the group spends working toward those goals and the level of persistence expended when group efforts fail to yield desired results [12].

The notion that shifting away from an existing, undesirable behaviour is predicated on a critical mass of group members believing that enough members will cooperate in enacting the new behaviour is well established [1,14,15]. This implies group goal selection. Perceptions regarding CE influence group goal selection and performance [16,17,18,19]. In fact, evidence suggests that perceptions regarding efficacy are better predictors of behaviour than prior performance or goal attainment [12].

CE perceptions are influenced by personal attributes, collective dynamics, and situational contexts [12,20]. As such, the mechanisms through which CE operates may differ for men and women. In many societies, existing psychosocial and structural inequalities translate to important differences in the social opportunities men and women have to engage with certain formal and informal community structures [21,22,23,24]. These differences are well cited, and have been known to create disparities between men and women in terms of their social inclusion, mobility, civic engagement and associational participation, and position within the communities in which they live [24,25,26]. These disparities may translate to differences in the mechanisms through which CE operates among men and women, such as the size and strength of their respective social networks, their sense of belonging or social attachment, and perceptions regarding the organisation and responsiveness of the community. In addition, various “gendered structures of constraint” may differentially influence perceived individual- and community-level psychological capabilities among men and women [22,26]. For example, normative expectations and social restrictions that preclude certain types of individuals (e.g., women) from moving and socialising freely outside of the home and within certain community groups may contribute to disparities among men and women in terms of their perceptions regarding self- and collective efficacy [27,28,29]. Consequently, existing evidence supports the idea that differences in collective action exist between genders [24].

In public health, and the WASH sector more specifically, we are still seeking to elucidate how complex, group-level factors effect behavioural outcomes and health impacts at individual and collective levels. While social constructs have been explored more broadly when examining various aspects of shared and communal WASH resources [30,31,32], investigations into collective efficacy in the context of community-based interventions remain scarce. Agentic factors such as self- and collective efficacy are featured extensively in behaviour and behaviour change theory [12,13,33,34]. Yet, only a limited number of studies have used empirical evidence to critically assess collective efficacy, and investigate its underlying structure (i.e., the relationships between the sub-constructs, such as factors and facets that are important to the measure of the construct) [19,35,36].

Researchers studying violent crime in urban Chicago, Illinois, USA found that two sub-constructs—social cohesion and trust among neighbours, and informal social control (i.e., neighbours’ willingness to intervene on behaviour for the common good)—define CE as a latent construct [19]. A study conducted in Blacksburg, Virginia, USA suggested that CE related to community computing was comprised of four sub-constructs—activism, informedness, belonging, and association [35]. Among teachers in elementary schools within one urban school district in the USA, CE was found to have a single factor structure that contained items tapping to group competence and task analysis [36]. Findings from these studies are conflicting, and all known psychometric examinations of CE have been conducted on data collected from literate populations in high-income contexts. No known studies have developed and applied CE measurement scales in such a manner that allows for a more thorough examination of the role CE plays in behaviour change and the overall effectiveness of community-based interventions operating in developing contexts. 

To address this gap, our team designed a series of studies to develop, validate, and employ scales to assess CE and compare perceptions between genders. For this particular study, we aimed to develop a metric that could be used in the context of a WASH trial, to assess pre-intervention behavioural control perceptions at baseline, and evaluate the effect of CE factors on the uptake of a demand-side sanitation and hygiene intervention at endline. We hypothesise that CE is an important antecedent of the cooperative behaviour and collective action needed to bring about sustained adoption of improved WASH practices at the level required to realise health impacts. Therefore, we used a systematic and widely accepted scale development and validation process [37] to develop and test CE measurement scales, the methods and results of which we present herein. We set out to investigate several research questions related to the measurement of collective efficacy, its constituent sub-constructs, and its factor structure. First, we were interested in elucidating which sub-constructs (e.g., constituent domains, factors/dimensions, facets) were salient for measuring collective efficacy, and the structure thereof, in the rural Ethiopian context. Second, we wanted to examine whether the psychometric characteristics of the resulting CE measurement models were compelling in terms of their ability to demonstrate construct validity, or the degree to which the scale measures what it purports to measure. Finally, we aimed to determine whether there were important differences in the measurement of CE between Ethiopian men and women. The development of these CE scales will allow for enhanced intervention design and targeting, and further examination of potential associations between CE, as measured by our scale, and intervention effectiveness, as measured through changes in WASH-related behavioural outcomes and health impacts.

We set forth three related hypotheses to test as we explored our research questions. First, we hypothesised that the factor solutions derived from our empirical data would support our theorised construct dimensionality. Second, we hypothesised that the CE measurement models produced via factor analytic methods would demonstrate high construct validity. Third, we hypothesised that given their status, mobilisation, and inclusion within their communities, men would have higher perceptions of behavioural control and related factors than women; leaders would have higher perceptions than non-leaders.

## 2. Study Overview

This study took place in Amhara National Regional State, Ethiopia as a sub-study of a larger cluster-randomised controlled trial entitled *The impact of enhanced, demand-side sanitation and hygiene promotion on sustained behavior change and health in Amhara, Ethiopia*, or *Andilaye* for short (registered on clinicaltrials.gov, NCT03075436).

### 2.1. Ethical Approval

The *Andilaye* trial and its sub-studies received ethical approval from Emory University’s Institutional Review Board (IRB00076141), the Amhara Regional Health Bureau Research Ethics Review Committee (HRTT0135909), and London School of Hygiene & Tropical Medicine’s Observational/Interventions Research Ethics Committee (Ref. 9595). Fieldworkers provided study participants with full details regarding the study prior to inquiring about consent to participate, and took steps to ensure confidentiality for all study participants.

### 2.2. Data Collection and Analysis

We used a five-step, sequential exploratory and confirmatory approach [38] to develop, refine, and validate a theoretically grounded and evidence-based CE measurement scale [37]. This process relied heavily on a factor analytic approach. Factor analysis is a psychometric method that allows for the measurement of latent constructs that cannot be directly observed or measured. In a factor analytic framework, latent constructs are measured through the analysis of manifest variables (e.g., survey items), indicators that represent certain aspects of the latent construct [39]. The analyses of these data can elucidate the underlying structure of the construct and its constituent sub-constructs. 

Factor analysis comprises a suite of analytical methods, including exploratory factor analysis (EFA) and confirmatory factor analysis (CFA). EFA is a descriptive analytical method used to determine the number of common factors in a measurement model, and identify which measured variables are indicators of the latent construct (i.e., identify factor structure) [40]. CFA is an evaluation approach that allows for direct testing and validation of hypothesised factor structures to assess their appropriateness as measurement models (i.e., determine construct validity, falsify hypothesised models). These types of analytical methods use matrix algebra to generate statistics used to reveal a construct’s underlying factor structure [41].

Below, we provide details related to each step of our scale development and validation process. Given the progressive nature of this process, we present methods and results for each step, in chronological order, followed by a discussion and conclusions. The methods and results presented below demonstrate the process by which we produced three refined CE measurement scales that demonstrated good construct validity.

## 3. Collective Efficacy Scale Development and Validation—Methods and Results

### 3.1. Step 1. Defining Collective Efficacy via a Hypothesised Framework

#### 3.1.1. Step 1. Methods

We leveraged theory and evidence to operationally define CE, and establish a hypothesised CE framework [37,42]. This began with a desk review of the literature during which we identified and extracted information regarding the CE construct and constituent sub-constructs. We then performed an applied thematic content analysis to re-organise emergent sub-constructs into key domains, dimensions/factors, and facets to generate the framework. We constructed definitions for each CE dimension/factor. In factor analytic terms, this step involved establishing operational definitions for the latent variables [43], and providing substantive justification for the hypothesised framework [44].

#### 3.1.2. Step 1. Results

We established the following operational definition for collective efficacy: A latent construct comprised of a combination of the cognitive and socio-structural components that facilitate a community’s shared belief in its ability to come together and execute actions related to a common goal. This conceptualisation of CE is grounded in evidence and theory, including Social Cognitive Theory and Social Learning Theory [19,34,45]. The resulting hypothesised CE framework represented a seven-factor conceptualisation, with items tapping to social disorder, social response, social capital, social equity, common values, community attachment, and agency. We hypothesised that these seven factors represented aspects of three domains: informal social control, social cohesion, and behavioural control (Table 1).

### 3.2. Step 2. Designing the Collective Efficacy Survey

#### 3.2.1. Methods

Next, we generated an item pool by extracting relevant prompts from existing surveys instruments [37]. We prioritised pre-existing, validated tools, when possible. We then coded survey items against our set of CE sub-constructs, removed repetitive items, and designed new items when no relevant prompts existed for a given sub-construct.

We re-structured all survey items such that they worked well with a five-point, Likert type response format [37]. Once our draft tool was developed, it was translated into Amharic, and back-translated into English to ensure the quality of the translations. During December 2016-February 2017, the CE survey was then piloted and iteratively refined through a series of formative research activities, which included two rounds of cognitive interviews and one round of focus group discussions. These formative research activities were conducted in Amharic in the Bahir Dar Zuria district (*woreda*) of Amhara National Regional State, Ethiopia. Interviews and group discussions were audio-recorded and transcribed directly into English.

Trained fieldworkers conducted four in-depth interviews (three women, one man) that employed think-aloud and verbal probing techniques [46] during an initial round of cognitive interviews. This cognitive validation process allowed us to ensure consistency between what we intended to convey through the survey items and participants’ understanding regarding the meaning of the survey items (i.e., assess face validity). This process helped us confirm that each survey item tapped to the desired sub-construct within our CE framework. Furthermore, our inclusion of cognitive interviews as a formative scale development activity allowed us to investigate whether there was early evidence regarding the substantive aspects of construct validity [47].

We then conducted four focus group discussions (two with women, two with men) to explore concepts and real-world examples related to our hypothesised CE framework. Based on our qualitative findings, we revised survey items to be more contextually relevant, and designed additional items to ensure each CE sub-construct was adequately measured, and our CE survey had reached conceptual saturation. Finally, we conducted a second round of cognitive interviews (four women, three men) to pilot this revised CE survey and again check participants’ understanding of the survey items.

#### 3.2.2. Step 2. Results

The formative survey and scale development work conducted during Step 2 resulted in a 50-item CE instrument. The 50 items were comprised of group-referent statements about interpersonal and ecological aspects of the respondent’s community that related to CE as well as self-referent statements about the respondent’s own sense of self, agency, autonomy, and level of engagement within his/her community. The content of the measured variables (i.e., items) used to operationalise CE is provided in Table A1. There, we provide detailed information on all 50 CE survey items.

### 3.3. Step 3. Administering the Collective Efficacy Survey

#### 3.3.1. Step 3. Methods

We trained fieldworkers to administer the CE survey by reading each survey item, followed by each of the five response options (i.e., completely disagree, partially disagree, neither agree nor disagree, partially agree, completely agree). Through two waves of data collection conducted during March–June 2017, fieldworkers visited *Andilaye* study households with the goal of administering the same 50-item CE survey to one man and one woman in randomly selected households. During the initial wave of data collection, the primary female caregiver of the trial’s index child (i.e., youngest child aged 1–9 years) was targeted for *Andilaye’s* baseline survey. Half of these respondents were randomised to receive our CE survey module. During the second wave, we targeted *Andilaye* study male heads of household. All men were targeted for the CE survey regardless of which survey the respondent from the initial wave of data collection was randomised to receive. This subsequent data collection round was designed to help us examine similarities and differences of CE perceptions between men and women in general, and household-level male-female dyads within a sub-set of study households in particular. The sub-set of dyadic households represented those in which: (1) a woman responded and was randomly allocated to the CE survey during the first wave of data collection; and (2) a man was willing and able to participate in the CE survey during the second wave of data collection.

#### 3.3.2. Step 3. Results

Fieldworkers targeted 1849 CE surveys. Consent was provided by 1846 respondents (i.e., 99% response rate). Fifteen observations were dropped due to data entry errors. The final analytical dataset contained 1831 observations; 1105 from men, 726 from women. At least one individual from 1311 households in 50 sub-district (*kebele*) clusters responded to our CE survey. We obtained CE data on household-level male-female dyads from 520 ‘dyadic’ households. Data were also obtained from 585 men and 206 women residing in 791 ‘non-dyadic’ households. See Table 2 for data on respondent demographics and household characteristics.

### 3.4. Step 4. Performing Psychometric Analyses

#### 3.4.1. Step 4. Methods

##### Data preparation and screening

Initial data cleaning and descriptive analyses were performed in Stata (version 15.0 StataCorp, College Station, TX, USA). We performed subsequent descriptive and all factor analyses (EFA, preliminary CFA, single-group CFA of EFA-derived factor solutions, multiple-group CFA and Multiple Indicators Multiple Causes [MIMIC] modelling) in Mplus software (version 8 Muthén & Muthén, Los Angeles, CA, USA).

To prepare our data for analyses, we first partitioned our CE dataset by gender, and then employed a random-number seed to identify two separate random split-halves for both men and women sub-samples. We designated one random split-half sample for scale development via EFA for each gender; the remaining random hold-out sample was reserved for scale validation via CFA of the EFA-derived factor solutions. This division of the dataset resulted in four split-half samples, two for women, and two for men (i.e., n_W1_, n_W2_; n_M1_, n_M2_). Univariate analyses performed in Stata and verified results in Mplus examined respondent/household characteristics and item distributions (frequencies and proportions—Table S1) for all 50 CE items on an aggregate level and between genders. We performed Mann-Whitney Rank Sum tests to determine whether there were any significant differences in respondent and household-level characteristics between split-halves.

##### Preliminary confirmatory factor analysis of the hypothesised CE framework

We decided *a priori* to first test our hypothesised CE framework (Table 1) via a preliminary CFA [48]. Poor model fit statistics for this preliminary CFA would signal that the hypothesised CE framework may need modification in order to produce an appropriate CE measurement framework. In the event model fit statistics indicated poor fitness, we decided *a priori* that we would perform EFA to determine alternative CE factor structures derived from our own empirical data, and conduct CFA again to test and validate the resulting EFA-derived factor structures [40]. 

For preliminary CFA, we used a robust weighted least-squares with mean and variance adjustment (WLSMV) estimation method [49] based on assessments of polychoric correlation matrices [50,51]. A sandwich estimator was applied to adjust for non-independence of observations within 50 *kebele* clusters. Because it would have been justifiable to conclude our analyses with CFA if the complex preliminary CFA indicated good model fit, we performed these analyses on the full men and women sub-samples (n_M_ and n_W_, respectively). We examined goodness-of-fit indices, assessing both absolute fit (e.g., χ^2^:df ratio, root mean square error of approximation [RMSEA]) and incremental, or relative fit (e.g., comparative fit index [CFI], Tucker-Lewis index [TLI]). Standard thresholds of acceptable and good model fitness were employed (i.e., χ^2^:df ratio < 3.0; RSMEA of ≤0.10 acceptable fit, ≤0.05–0.06 good fit; CFI & TLI ≥ 0.90 acceptable fit, ≥0.95 good fit) [40,52,53]. Factor loadings less than 0.32 were considered non-salient (i.e., not statistically meaningful) [54]. *Post hoc* refinements included the deletion of items with non-salient (factor loadings < 0.32) and/or non-significant (two-tailed *p* > 0.05) factor loadings. We also dropped all factors with less than three items with salient and significant factor loadings, as these factors may have insufficient component saturation, meaning the factor may not have been fully conceptually explained by the emergent items, which could compromise factor interpretation [48].

##### Exploratory factor analysis

We performed complex EFA on one split-half of data from both men and women sub-samples (n_W1_ and n_M1_, respectively). As with preliminary CFA, we used a robust WLSMV estimation method based on assessments of polychoric correlation matrices for EFA, and applied a sandwich estimator to adjust for non-independence [49,50,51]. An oblique rotation was indicated due to hypothesised item correlation, and Promax was selected *a priori* as the specific oblique rotation method for these analyses.

Decision rules related to factor retention were based on a combination of: (1) mathematically based and heuristic descriptive guides (i.e., Kaiser-Guttman rule (eigenvalue > 1.0) [55], scree-plot); (2) goodness-of-fit; and (3) other substantive justification, such as results from cognitive interviews, and theoretical and empirical evidence [40,44,48]. As with preliminary CFA, we employed a holistic approach to evaluate goodness-of-fit indices for EFA. The same thresholds used for preliminary CFA were used for EFA, but we also included an assessment of root mean square residual (RMSR); values below 0.08 indicate reasonable model fit [52].

Oblique rotations produce pattern coefficients that do not fully characterise the relationship between an item and a given factor [44]. Therefore, in order to appropriately interpret EFA results, we evaluated both the factor pattern and factor structure matrices [48]. Structure and pattern coefficients with an absolute value greater than 0.32 were considered salient. Items with factor loadings less than this threshold poorly measured the latent factors, and were eliminated in a step-wise manner [54]. We iteratively re-analysed measurement models subsequent to item reduction [44]. To be retained, factors needed to demonstrate adequate component saturation and sufficient evidence that they were at least adequately measured (i.e., at least three items with factor loadings greater than 0.32, and no or limited item cross-loadings) [56]. Only complex variables (i.e., those with salient factor loadings on more than one factor [cross-loadings]) with strong substantive justification for their cross-loadings were retained. Models that represented the most readily interpretable (i.e., the simplest solution, per Thurstone criteria [57]—outlined in the Supplemental Material) and theoretically justifiable solutions were selected for the refined, gender-specific factor solutions [44].

With regard to the interpretation of EFA results, factor loadings indicate the pattern of item-factor relationships, and are often referred to as pattern coefficients [40]. Factor loadings represent completely standardised estimates of regression slopes for predicting the indicators from the latent variable [40]. While some methodologists caution against the use of thresholds, common guidelines for the interpretation of factor loadings can be used to facilitate interpretation of results (e.g., factor loadings >0.71 excellent, >0.63 very good, >0.55 good, >0.45 fair, and >0.32 adequate) [54]. 

##### Single-group confirmatory factor analysis of EFA-derived, gender-specific factor solutions

During CFA of EFA-derived factor solutions, we used split-half hold-out samples (n_W2_, n_M2_) to validate EFA-indicated, gender-specific measurement models. The underlying structure used to operationalise the latent factors were those indicated in the factor solution produced via EFA. We identified the scale of every latent factor through the use of marker indicator items, which we identified as the item that demonstrated the highest factor loading on its respective factor, per EFA results [40]. As with our preliminary CFA and EFA, we performed these CFAs using WLSMV with a sandwich estimator to adjust for non-independence. Through *post hoc* model refinements, we eliminated items with non-salient (i.e., factor loading < 0.32) and/or non-significant factor loadings.

We used the same process for holistically examining goodness-of-fit and carrying out *post hoc* model refinements for the CFA of EFA-derived models as those employed during the preliminary CFA. After examining fit statistics, we assessed residuals and modification indices for indications of localised areas of strain (i.e., misfit) in the measurement models. Modification indices greater than 3.84 indicated opportunities for further model refinement and fit improvement, through the estimation of additional parameters, if justified [40,58].

#### 3.4.2. Step 4. Results

##### Preliminary confirmatory factor analysis of hypothesised CE framework

Results from the preliminary CFA of our hypothesised CE framework indicated that the men’s refined model (i.e., with *post hoc* adjustments) demonstrated moderately acceptable absolute fit, but poor incremental or relative fit (χ^2^:df ratio = 2.606, RSMEA = 0.038 [0.036–0.040], CFI = 0.911, TLI = 0.904). This suggested that while our hypothesised CE framework represented a plausible structure of the mechanisms through which the CE process operates among men in the Ethiopian context, an alternative framework may have provided a better measure of CE. The women’s model did not fit the data well (χ^2^:df ratio = 3.409, RSMEA = 0.058 [0.055–0.060], CFI = 0.895, TLI = 0.888), which indicated that the data failed to validate the hypothesised CE framework for women respondents. This suggested that the CE framework required modification in order to reveal the mechanisms through which CE operates for women in rural Ethiopia. See Table A2 and Table A3 for additional preliminary CFA results. These findings provided rationale for performing EFA.

##### Scale development and validation samples, balance of respondent and household characteristics

Our split-half EFA samples consisted of 366 observations from women (i.e., participant to item ratio of over 7:1), and 555 observations from men (i.e., participant to item ration of 11:1). While the participant to item ratio was lower for women, split-half sample sizes were sufficient for both genders according to standard guidance [48,54]. All respondent demographics and household characteristics were balanced across aggregate and gender-specific sub-samples (results not displayed).

##### Factor extraction and item reduction

Seven factors were extracted during final EFA for both gender-specific EFA-derived factor solutions. We present factors and items indicated in both men’s and women’s EFA-derived factor solutions in Table 3 and Table 4, respectively. Information related to the item reduction processes for both models, and a detailed summary of each factor emerging from the gender-specific EFA-derived models are provided in the Supplemental Material. Below, we summarise the resulting gender-specific EFA-derived measurement models, by domain.

##### Men’s collective efficacy measurement model

EFA results revealed a seven-factor men’s CE measurement model with good model fit (χ^2^:df = 1.209, RMSEA = 0.019, RMSR = 0.037). The seven factors included social response, social networks and personal agency, social attachment, common vision, community leadership, associational participation, and community organization. Social response corresponded to the informal social control domain, though it also tapped to certain aspects of cognitive social capital (e.g., trust in community members, reciprocity of knowledge) that may influence social response. The social networks and personal agency factor corresponded to the cognitive social capital domain, though it also tapped to structural social capital, as it reflects the strength and responsiveness of one’s social structures. Social attachment and common vision factors corresponded to the social cohesion domain. Community leadership and associational participation factors pertained to the structural social capital domain. These factors and the concepts reflected in their constituent items align with our hypothesised operational definitions of informal social control, social cohesion, and behavioural control (Table 1 and Table A4). See Table 3 for information regarding the specific items that tapped to the CE factors in the men’s CE measurement model. Modification indices above 3.84 were all relatively low, meaning localised strain was relatively low in all areas identified. No further modifications were deemed theoretically or mathematically justifiable. The standardised estimates of factor loadings from this model were acceptable (Table 3). 

After dropping one item (ADVICE) from the male EFA-derived factor solution as a result of less than minimal variance, we conducted CFA on the remaining items tapping to seven factors. *Post hoc* model refinements yielded a refined 31-item, seven-factor solution. The refined CFA model validated the EFA-derived model with minor modifications, and demonstrated good absolute and incremental model fit (χ^2^:df ratio = 1.498, RSMEA = 0.030 [0.025–0.035]), CFI = 0.971, TLI = 0.968). The vast majority of items (87%, 27 of 31) comprising the refined solution demonstrated very good to excellent factor loadings (i.e., loadings > 0.630) on a single factor (Table 3).

##### Women’s collective efficacy measurement model

EFA results revealed a seven-factor women’s CE measurement model with good model fit (χ^2^:df = 1.281, RMSEA = 0.028, RMSR = 0.041). The seven factors included social networks and reciprocity, social disorder, social attachment and personal agency, social response, common vision, associational participation, and community organisation and leadership. The social networks and reciprocity factor corresponded to the cognitive social capital domain, though it also tapped to certain aspects of structural social capital, as it reflected perceptions related to collectives of individuals that promote and protect mutual or personal interests. The informal social control domain included factors related to social disorder and social response. Two factors, social attachment and personal agency and common vision, comprised the social cohesion domain. Associational participation and community organisation and leadership corresponded to the structural social capital domain. These factors and the concepts reflected in their constituent items align with our hypothesised operational definitions of informal social control, social cohesion, and behaviour control. See Table 4 for information regarding the specific items that tapped to the CE factors in the women’s measurement model.

We conducted CFA on the items tapping to seven factors, as indicated by EFA. *Post hoc* model refinements yielded a refined 33-item, six-factor solution. The refined CFA model validated the majority of the EFA-derived factor structure, and demonstrated adequate model fit (χ^2^:df ratio = 1.574, RSMEA = 0.040 [0.034–0.045]), CFI = 0.962, TLI = 0.958). A majority of items (79%, 26 of 33) on the refined CFA solution demonstrated very good to excellent factor loadings (i.e., >0.630), and 12% (4 of 33) demonstrated good factor loadings (i.e., between 0.550–0.629) (Table 4).

##### Comparison of men and women’s CE models, identification of a parsimonious model

As indicated in Table 5, there was considerable overlap between gender-specific CE measurement models. We therefore identified a parsimonious CE scale that reflected those items included in both men’s and women’s refined CE measurement models (Table 5). These findings suggest that the mechanisms and processes through which CE operates are similar between men and women in rural Ethiopia, but key differences exist, particularly with regard to the number and nature of constituent factor items (Table 5). The gender-specific models represent more saturated, and slightly better fitting models (Table A5). Those models allow for exploration into specific mechanisms through which CE specifically operates for men and women, respectively.

A subsequent CFA that tested the fit of the men’s and women’s data to the parsimonious CE measurement model [44] demonstrated good model fit (Table A5). These results suggest that the two gender-specific, saturated models and the parsimonious model all demonstrated construct validity, suggesting they are appropriate metrics for measuring CE.

### 3.5. Step 5. Testing Hypotheses

#### 3.5.1. Step 5. Methods

##### Multiple-group CFA and MIMIC modelling for assessment of differential item functioning

We performed multiple-group CFA to examine certain aspects (e.g., factor loadings) of measurement invariance between genders. Measurement invariance means that the probability of selecting a given item response category is comparable across groups, given similar levels of the latent construct being measured [59]. This multiple-group CFA differed from previous single-group, gender-specific CFA in that we were able to simultaneously employ input matrices from both men’s and women’s datasets. Due to the relatively small proportion of households with leadership status, we did not perform multiple-group analyses on this variable.

Next, we performed Multiple Indicator Multiple Causes (MIMIC) modelling to test the validity of our parsimonious CE measurement model in the presence of other relevant covariates, and assess differential item functioning (DIF) [60]. DIF, or measurement *non*-invariance occurs when people from different groups (e.g., men, women) with similar levels of the latent construct have different probabilities of responding to an item in a certain way [61]. Our structural equation MIMIC models consisted of a measurement model component reflected by the refined parsimonious CE model, and a structural model component that specified the direct effects of gender and household leadership covariates on latent factor variables and relevant item indicators. Significant direct effects would indicate DIF between men and women respondents.

The same validation sub-samples used for single-group CFA were used for these analyses, but we aggregated gender-specific sub-samples (n_2_). As we constructed our MIMIC models, we first established baseline models that introduced gender and leadership status covariates, but assumed no direct effects of the covariates on any individual CE items. Then, we employed a step-wise, forward selection approach to assess direct effects between these covariates and relevant item indicators. We examined the modification indices, and identified the item indicator with the highest significant, meaningful, and substantively justifiable modification index. We added a direct path between the identified item indicator and relevant covariate. We employed the DIFFTEST option in Mplus to assess whether the additional direct path improved model fit. Given we had a relatively large sample size, it was likely that DIFFTEST statistics would be significant [62], so we evaluated and compared other model fit indices as well.

##### Collective efficacy factor score calculation

We used two coarse CE factor score calculation methods (i.e., non-refined, un-sophisticated procedures) to generate both average and weighted average CE factor scores [63]. Higher factor scores represented higher levels of perceived behavioural control over the respective CE factors. We generated CE factor scores for each respondent by summing his/her responses across all items in each factor (i.e., 1 = completely disagree, 2 = partially disagree, 3 = neither agree nor disagree [neutral], 4 = partially agree, 5 = completely agree), and dividing that sum by the number of items tapping to the factor. This approach, however, assumes that all items have the same level of influence, or measurement proximity to their respective latent factor. We have demonstrated that this is not the case. We generated scores in this format to allow for easy comparison of scores, should the tool be used in different contexts. This is not only a simpler method compared to context-specific weighting, but also allows for more appropriate comparison of results outside of the dataset in which the weights were derived [63]. We also calculated weighted average CE factor scores, for which a weight that was equivalent to the item’s factor loading was applied to each item score prior to the generation of the average (weighted) factor score. We examined whether there were statistically significant differences in factor scores by gender and leadership status, and between household-level dyads via a regression-based approach with cluster robust standard errors to adjust for within-village clustering. For dyads, we regressed pair-wise factor score differences with cluster robust standard errors [64].

#### 3.5.2. Step 5. Results

##### Multiple-group confirmatory factor analysis and MIMIC model results

Multiple-group CFA indicated that factor loadings between men and women were similar (Table 6). We present model fit statistics, unstandardised and standardised beta estimates, and standard errors for competing MIMIC models in Table A6. The baseline MIMIC model with latent variables regressed on gender and household leadership covariates, but no direct effects between item indicators (i.e., Model 3 in Table A6) demonstrated good model fit (χ^2^:df ratio = 2.124; RMSEA [90% CI] = 0.035 [0.032–0.039]; CFI = 0.965; TLI = 0.960). Only two items from the baseline MIMIC model with latent factors regressed on gender and household leadership status demonstrated modification indices above 3.84. The HARMONY item indicator had the highest, albeit relatively small, modification index (14.342) on gender. This finding indicated that there was DIF between men and women for this item, so we added a direct path between HARMONY and gender.

On the model iteration specifying this direct path (i.e., Model 4 in Table A6), both the unstandardised B and standardised β were salient (i.e., −0.457 and −0.448, respectively), indicating that men scored HARMONY, on average, 0.45 units lower than women. This refined model fit the data well (χ^2^:df ratio = 2.090; RMSEA [90% CI] = 0.035 [0.031–0.038]; CFI = 0.966; TLI = 0.961), and DIFFTEST statistics indicated that model fit improved with the inclusion of this direct effects parameter. The modification indices of the resulting model indicated only one additional item with a low modification index (ACTEXOGP, 3.911), and small but non-salient direct effects. Therefore, no further model refinements were made. The final MIMIC model accommodated uniform DIF by incorporating a direct effect between gender and HARMONY, and indirect effects of gender and household leadership status covariates on factor means.

##### Final collective efficacy measurement metrics 

To avoid DIF and ensure construct validity, we dropped HARMONY from CE measurement model, and re-ran a final CFA [65]. As demonstrated in Table 6, the validated model indicated good fit (χ^2^:df ratio = 2.191; RMSEA [90% CI] = 0.036 [0.033–0.040]; CFI = 0.966; TLI = 0.960), with all items loading significantly and saliently a single factor. The final, validated parsimonious CE scale included 26 items tapping to six factors: social response, social networks and personal agency, social attachment, common vision, associational participation, and community organization and leadership.

##### Assessment of collective efficacy scores across cohorts

**Figure 1:**
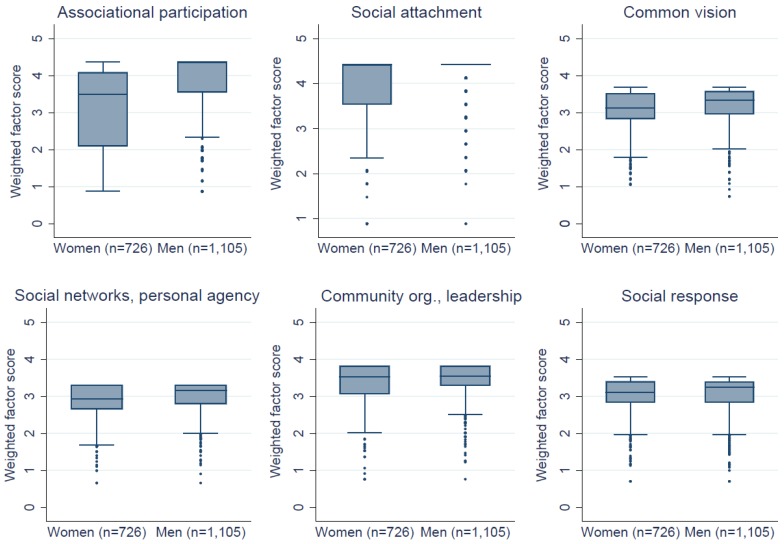
Collective efficacy factor scores (weighted), by respondent gender. Factor scores are visualised as box plots, which depict the distribution of the data through quartiles. The boxes represent the inter-quartile range (i.e., 25% and 75% quartiles comprise the outer edges of the boxes, while the median is indicated by the line inside the box). The lines that extend vertically from either side of the box (i.e., whiskers) indicate the variability of the data outside the upper and lower quartiles. Outliers are plotted as individual points.

Overall, men scored all CE factors significantly higher than women (Figure 1), suggesting men have higher behavioural control perceptions than women. Men and women in household-level male-female dyads scored all CE factors significantly different, with the exception of social response (β_unweighted_ = 0.07, [95% CI: −0.02, 0.16]; β_weighted_ = 0.05, [95% CI: −0.02, 0.11]). This indicates CE perceptions may differ significantly within households. Factor scores only differed significantly between respondents with leadership roles and those without on two factors: social networks and personal agency (β_unweighted_ = 0.10, [95% CI: 0.03, 0.17]); β_weighted_ = 0.07, [95% CI: 0.02, 0.12]), and associational participation (β_unweighted_ = 0.15, [95% CI: 0.02, 0.28]; β_weighted_ = 0.13, [95% CI: 0.02, 0.24]).

## 4. Discussion

This study contributes to the development of a metric that can be used in community-based health and development programmes, to inform intervention design, identify communities ripe for programmatic targeting, and diagnose factors related to intervention effectiveness. Such a metric may be useful for any programme that targets collective behaviours. It may be particularly beneficial for the WASH sector, as differentials in CE factors may help explain poor uptake of community-based WASH interventions, regression to unimproved behaviours, and lower than expected health gains. 

The structures derived through our factor analytical approach reflect rigorously derived measurement models that are grounded in theory and evidence-based. Findings from our exploratory analyses suggested that CE is a complex, multi-dimensional social construct. EFA-derived factor structures suggest that social response, social networks, social attachment, common vision, associational participation, and community organization and leadership are important factors in the measure of CE among men and women in rural Ethiopia. These factors reflect domains related to social cohesion, informal social control, and cognitive and structural social capital.

Elucidating these CE factors and examining their constituent sub-constructs is important to consider for the design of intervention content and development of implementation strategies. In terms of content, failing to acknowledge and address these CE factors as part of community-based intervention approaches may be problematic for the uptake of such interventions. For instance, a lack of common vision may prove to be a barrier to communities accepting WASH facilities coverage and use targets as communal goals. If the issue of common vision is not addressed alongside mainstream WASH intervention activities, the intervention may fail to stimulate the collective action and cooperative behaviour necessary to achieve programmatic goals at the community level. When collective goals are set, progress toward them may be inhibited in the presence of inadequate or insufficiently nurtured supporting social networks, which may limit the diffusion of innovations within a community [66]. It may be beneficial for interventions to leverage and strengthen social networks to facilitate uptake of improved WASH practices while also addressing personal agency and social inclusion of women within community structures. Addressing these CE factors within the context of community-based interventions may therefore create an environment more conducive to engendering and maintaining positive change. In terms of implementation strategies, it is important to consider CE factors and differentials in factor scores when determining at whom interventions are targeted, and through which mechanisms they are being delivered. Interventions that target programme participants who have low perceptions of self- and collective agency, and are not well positioned to serve as change agents within their households, social networks, and community may prove to be ineffective. Poor associational participation among women may impede adoption and maintenance of improved WASH behaviours, particularly those aimed at infants and young children, when interventions are delivered through community associations or groups. Other mechanisms and intervention techniques, such as one-on-one skills-based household counselling visits may serve to better facilitate behavioural uptake while also enhancing action knowledge and improving self-efficacy perceptions in contexts where women do not readily engage with community groups. Several CE factors may need to be addressed through different types of intervention techniques, which should be considered from the outset of intervention design.

The CE structures revealed by our EFA analyses differed slightly from our hypothesised framework. For example, in our hypothesised CE framework, we conceived that agency would emerge as an independent CE factor/dimension encompassing items related to individual- and collective-level perceptions. However, results from our factor analyses indicated that these agentic concepts are closely tied to social networks and social attachment for men and women, respectively. These findings may suggest that agency perceptions are constituent influencers of these sub-constructs as opposed to being important stand-alone CE factors. On the other hand, agency may not have emerged as an independent factor in our EFA given some aspects of autonomy control beliefs, motivational commitment to communal goals, resilience to adversity, and performance accomplishments were not fully represented within the CE survey. The literature suggests an array of conceptual definitions for CE and its constituent sub-constructs, and our EFA-derived factor structures did not deviate considerably from our hypothesised framework. Therefore, we found sufficient theoretical justification to support our refined, EFA-derived factor structures.

Our EFA exposed some key differences in gender-specific factor structures. We used a two-pronged approach to compare CE among men and women by first examining gender-specific CE mechanisms via single-group measurement models, and then assessing perceptions of common CE factors between genders via comparison of factor scores. One of the most notable distinctions with regard to underlying CE mechanisms (i.e., factor structures) was that personal agency was linked to different sub-constructs in the two gender-specific measurement models. These findings suggest that for women, one’s sense of self-agency is linked to one’s sense of belonging or social attachment, while for men, it is linked to expectations regarding the responsiveness of one’s social networks. This may be a result of a higher level of social inclusion, mobility, and ability of men to engage more readily with both formal and informal community structures in the rural Ethiopian context. We also observed that community leadership emerged as its own CE factor in the men’s CE measurement model, while these items loaded to the “community organisation and leadership” factor in the women’s model. This may be an artefact of men having more access to and engagement with people holding such positions within the community.

Our scales produced quantitative measures of CE for all respondents in the form of factor scores for each CE factor. Higher scores reflected higher levels of perceived behavioural control over the respective CE sub-constructs. These findings revealed that, overall, men scored higher on all CE factors than women. This is notable not only for gendered WASH interventions that may be targeting female caregivers as their primary programme participants, but any community-based programme seeking to address gender and women’s empowerment, more broadly. For WASH interventions specifically, the sector should note that our findings suggest women may not inherently be in a position to influence adoption of promoted behaviours and practices readily among their peers, across all contexts. In order for such interventions to effectively engage women, they may need to address aspects of perceived agency and social inclusion alongside programme-specific objectives. These findings corroborate existing evidence [24,26,67] that women often suffer from various “gendered structures of constraint”, including limitations related to social inclusion, civic engagement, and membership and participation in community structures. CE factor scores suggest that differences in the social inclusion of men and women in rural Ethiopia appear to have created disparities in terms of their perceived individual- and community-level behavioural control perspectives.

We observed differences between men’s and women’s CE perceptions for a variety of reasons. Becoming aware of the disparities in CE factor scores should influence sampling methodology. Implementers interested in assessing CE and targeting or evaluating programmes that operate at a collective level should measure CE among both men and women to yield data that are representative of the larger programme population. Such a sampling approach will offer a more holistic, less biased appraisal of CE that accounts for heterogeneity of perspectives. This can be done through the employment of our parsimonious scale, which is suited for use among both men and women. CE scale results can be used to inform the design of community-based interventions. Findings from CE surveys may help identify the specific CE factors that need to be addressed, across genders, depending on an intervention’s target audience (e.g., women, men, both women and men). 

Although our results suggest a more comprehensive and complex underlying CE factor structure than previous studies, these findings do corroborate results from some of the existing literature. When considering the existing empirical evidence, certain sub-constructs seem to transcend contexts, languages, and culture when it comes to the measure of CE. Two of the factors proposed from a study examining CE in the context of community computing in Blacksburg, Virginia, USA [35]—belonging or social attachment and association (associational participation, in our case)—were also indicated in our refined gender-specific and final, parsimonious CE measurement scales. While activism and informedness, the two other factors identified through that study, did not emerge as factors in our analyses, items that represented these concepts were included in our refined gender-specific and final, parsimonious CE scales. Our findings suggest that social cohesion and informal social control domains proposed by authors of a study investigating CE and violent crime in urban Chicago, Illinois, USA [19] are important for the measure of CE, but do not necessarily manifest as factors themselves.

Our more comprehensive factor structures were substantively justified, and suggest that our CE scales include additional factors and items not included in other metrics. This is likely an artefact of the emphasis we placed on the activities conducted during the first two steps of our scale development process. To our knowledge, based on information provided in the literature, previous CE scale development studies did not include or heavily emphasise these scale development steps. As a result, our CE scales reveal more CE factors than the alternatives, and may therefore allow for a more accurate measure of CE and its effect on important behavioural outcomes at the community level. It seems fitting that a construct as complex as CE would need to draw on a more nuance underlying structure to ensure content validity.

This study had several procedural and analytical strengths and limitations. Our scale development and validation approach was a strength in that it reflected a mixed-methodological process that included focus group discussions and cognitive validation. We actively reflected on these qualitative data, which we used along with other theoretical and empirical evidence to make evidence-based, substantively justified modelling decisions. The size of our female sample may have been a limitation. While our gender-specific split-half sub-samples met common sample size guidelines, our sample size-dependent model fit statistics (e.g., χ^2^ *p*-value) indicated the sample sizes may have only been borderline sufficient for a factor structure as complex as those related to our CE measurement models. While our deliberation of possible factor models was heavily informed by substantive considerations, there is a dearth of existing theoretical and empirical evidence on collective efficacy in low literacy and resource poor settings such as those in which this research was carried out. Our own qualitative evidence aside, it is possible that decisions based on substantive considerations were not appropriate in these contexts. As such, the factor structures identified through our analyses may not necessarily generalise to other populations. Similarly, these findings may not translate to individuals and populations in which mental well-being is poor, and depression and anxiety are common, as these factors may interact with other behavioural determinants to influence behavioural control perceptions.

The external validity of our proposed CE measurement metrics, including their ecological (generalisability to other settings), population (generalisability to other people), and historical (generalisability over time) validity requires further examination [68]. We agree with our colleagues from the water insecurity sector that context-specific scales are advisable [69,70,71]. We recognise that the underlying structure of CE, as an inferential process, may differ substantially from context to context, as related sub-constructs are largely informed by context-specific political economies and social schemas. For instance, factors related to empirical and normative expectations regarding cooperative behaviour that likely inform perceptions regarding agentic concepts such as self- and collective efficacy are steeped in rich historical and cultural traditions may not be comparable on a global scale. In some contexts, women may be more integrated into endogenous community structures, so gendered differentials in associational participation, social networks, and perceptions regarding social attachment may be less pronounced in those settings. In environments such as India, where caste and class structures are important, different CE-related factors, such as social disorder may have a stronger influence on personal and collective-level behaviour control perceptions [72]. Findings from additional formative research and psychometric assessment efforts both in Ethiopia and elsewhere can; however, enhance our early CE work, further assess the validity of our CE scale, and reveal whether various CE sub-constructs transcend contexts.

## 5. Conclusions

Our CE scales offer new tools for the examination of collective behaviour factors. These tools can be used for programmatic targeting, intervention design, and diagnostic investigations into the role CE factors play in the uptake of community-based interventions and their impacts on health and development. They also facilitate the generation of evidence related to factors falling along the causal chain, which may explain why biologically plausible health gains are not being achieved by WASH interventions, as expected. Important differences in perceptions related to CE factors among men and women exist. These disparities should be acknowledged and addressed in the design of intervention content and implementation strategies for community-based interventions, particularly those promoting improved WASH behaviours for infants, young children, and their caregivers.

## Figures and Tables

**Table 1:** Hypothesised collective efficacy framework.

Domain	Dimension/Factor	Definition	Related Facets
Informal social control	Social disorder	General conflict and threats to the existing order—e.g., incivility	Incivility, intolerance, people not living in harmony
	Social response	Community members actively address social issues—e.g., respect differences, celebrate successes, and react to social inequity (below)	Willingness to intervene, community support in times of crisis, collective morals, tolerance, inter-group cooperation
Social cohesion	Common values	Community members share common values, beliefs and ideologies	Order, group cohesion and inclusion, social integration, acceptance, collective norms and ideals, common civic culture
	Social capital *	Residents have strong social networks within the community that establish a sense of trust among community members and leaders and allow for acts of reciprocity	Social networks and social capital, supporting networks and reciprocity, social organisation and groups, associational activity and common purpose, social trust, social bonds, social safety nets, trust and solidarity, volunteer activities
	Social equity	Residents have equal access to resources, services and opportunities within the community and there are safety nets in place in times of crisis	Social solidarity and reductions in wealth disparities, information and communication, contribution to household resources, social justice and equity, ownership of household assets/resources
	Community attachment	Residents feel a sense of connection to their community whether it is through ownership of resources/assets, through social ties or both. Being a part of the community is an aspect of a resident’s identity	Place attachment, place identity, sense of belonging
Behavioural control	Agency	Community members’ belief that they themselves are capable of achieving an identified goal (i.e., self-efficacy) Community members’ belief that their community as a whole is capable of making positive changes (i.e., collective action), and that they ought to be doing so. Here, we also explore community members’ perceptions regarding the need for exogenous intervention to achieve common goals	Perceived performance experiences (i.e., enactive mastery), vicarious experiences, physiological arousal (i.e., emotional state/control), self-esteem Collective action and cooperation, participation in collective action, collective behavioural control, empowerment

Note: * Including both structural components (e.g., civic structures engagement/participation) and cognitive components (e.g., trust, reciprocity).

**Table 2:** Respondent demographics, household- and cluster-level characteristics, by gender.

Characteristics	Aggregate		Men		Women	
Number of respondents	1831		1105		726	
Respondent demographics	*n* %					
Median age (IQR)	35	(29–45)	40	(31–47)	31	(27–38)
Relation to head of household						
Respondent is the head of household	1170	64%	1002	91%	168	23%
Spouse	540	29%	4	<1%	536	74%
Other relative	114	6%	92	8%	22	3%
Other non-relative	7	<1%	7	<1%	0	0%
Married	1667	91%	1014	92%	653	90%
Household-level characteristics	*n* %					
Median number of members per household (IQR)	5	(4–6)	5	(4–6)	5	(4–6)
Religion						
Orthodox Christian	1730	95%	1047	95%	683	94%
Muslim	55	3%	33	3%	22	3%
Other	41	2%	20	2%	21	3%
Head of household’s education (highest level attained)						
No formal education	1317	72%	801	72%	516	71%
At least some first cycle primary (grades 1–4)	175	10%	104	9%	71	10%
At least some secondary (grades 5–8)	233	13%	139	13%	94	13%
Any high school or above	91	5%	53	5%	38	5%
Refuse or do not know	15	<1%	8	<1%	7	<1%
Access to household latrine (any type)	1394	76%	854	77%	540	74%
Primary drinking water source location						
In compound	68	4%	40	4%	28	4%
Outside compound	1757	96%	1060	96%	697	96%
Household member with leadership in a community structure *	215	16%				
Total number of household-level male-female dyads *	520	40%				

Notes: IQR = inter-quartile range. Five observations from the men’s sub-sample (and therefore the aggregate as well) were missing data on the number of members in their households, the head of household’s highest educational attainment, religion, and ethnicity; one observation from the men’s sub-sample was missing data on marital status. Two observations from the women’s sub-sample were missing data on the number of members per household. * 1311 households.

**Table 3:** Factor loadings for random split-half samples for EFA and CFA of EFA-derived factor solutions, men.

Factors and Associated Items	Item	Final EFA—Factor Pattern Coefficients (n_M1_ = 555)	Final EFA—Factor Structure Coefficients (n_M1_ = 555)	Baseline CFA (n_M2_ = 550)	Refined ^†^ CFA (n_M2_ = 550)
Factor 1: Social response (average factor loading = 0.565; average structure coefficient = 0.605; average factor loading on refined CFA = 0.634)					
People in this community live in harmony with each other most of the time.	HARMONY	0.694	0.489	0.440 *	0.438 *
When there is a problem in this community, people come together to discuss how it should be solved.	COMPRSLV	0.654	0.702	0.692 *	0.690 *
People in this community can be trusted.	COMTRUST	0.634	0.741	0.683 *	0.682 *
If there is a problem that affects the entire community, for instance, crop disease, people in this community will help each other.	HLPCRPDZ	0.620	0.657	0.698 *	0.695 *
This is a close−knit community (i.e., people in this community have close personal relationships with each other).	CLOSE	0.592	0.720	0.791 *	0.789 *
Most people in this community have similar beliefs about what is right and what is wrong.	SIMBLIEF	0.567	0.484	0.409 *	0.408 *
If there is a big dispute between two persons, other people from the community will help in solving the problem.	SLVDISPU	0.510	0.596	0.652 *	0.649 *
People in the community share new knowledge with their neighbour if they learn something new.	SHAREKNO	0.414	0.649	0.726 *	0.724 *
Differences between people, such as the amount of land they own, often causes problems in this community.	DIFPROBS	−0.398	−0.403	−0.278 *	-
Factor 2: Social networks and personal agency (average factor loading = 0.588; average structure coefficient = 0.678; average factor loading on refined CFA = 0.668)					
I have the capacity to achieve my future aims.	SELFEFF	−0.985	−0.841	0.510 *	0.512 *
I have the ability to contribute to this community’s development.	SEDEV	−0.600	−0.729	0.675 *	0.674 *
If you suddenly need some money, you can borrow from a person or group in your community.	BORMONEY	−0.479	−0.595	0.759 *	0.758 *
If you and your relatives suddenly had to go away for a day/two, you could count on your neighbours to take care of your children.	NEICAREG	−0.477	−0.623	0.657 *	0.657 *
My neighbours sometimes come to me to share their problems and get help.	COME4HLP	−0.398	−0.602	0.737 *	0.738 *
Factor 3: Social attachment (average factor loading = 0.577; average structure coefficient = 0.723; average factor loading on refined CFA = 0.864)					
I feel attached to this community and its people.	ATTACH	0.795	0.836	0.860 *	0.857 *
People in this community accept me as a member of the community.	ACCEPT	0.673	0.792	0.863 *	0.864 *
Being a member of this community is part of who I am.	IDENTITY	0.631	0.779	0.871 *	0.871 *
People in this community should work together to develop the community.	SHOULDEV	0.455	0.637	0.075	-
People in this community have the capacity to make positive changes by coming together.	COLLEFF	0.332	0.571	0.071	-
Factor 4: Common vision (average factor loading = 0.519; average structure coefficient = 0.657; average factor loading on refined CFA = 0.733)					
Most people in this community have similar hopes about the future development of the community.	SIMHOPES	0.702	0.821	0.832 *	0.830 *
People in this community share the same ideas on how village matters should be managed.	COMMGMT	0.651	0.789	0.890 *	0.887 *
Most people in this community have common values, for example, they value hard work.	COMMVALU	0.586	0.697	0.813 *	0.809 *
People in this community have the capacity to make positive changes by coming together.	COLLEFF	0.496	0.652	0.702 *	0.760 *
During a crisis situation, such as drought, government services are distributed equally by the community to all households in need.	DISTCRIS	0.344	0.408	0.462 *	0.458 *
People in this community should work together to develop the community.	SHOULDEV	0.336	0.573	0.598 *	0.656 *
Factor 5: Community leadership (average factor loading = 0.590; average structure coefficient = 0.713; average factor loading on refined CFA = 0.720)					
Formal administrative leaders, like the *kebele* manager, provide support to this community.	ACTLDR2	0.871	0.814	0.721 *	0.720 *
This community’s leaders can be trusted.	TRUSTLDR	0.732	0.823	0.899 *	0.899 *
There are people in this community who show strong leadership.	UNOFLDRS	0.372	0.634	0.733 *	0.734 *
I typically accept advice from others in this community.	ADVICE	0.385	0.581	-	-
Factor 6: Associational participation (average factor loading = 0.702; average structure coefficient = 0.825; average factor loading on refined CFA = 0.913)					
I participate in activities held by any community−based associations, such as the Edir.	PARTCBGP	−0.792	−0.883	0.953 *	0.953 *
I attend meetings of a community−based association, such as the Edir.	ACTCBGP	−0.794	−0.897	0.960 *	0.960 *
I participate in activities held by any government or NGO−initiated community development group, such as the Development Army.	ACTEXOGP	−0.520	−0.694	0.828 *	0.827 *
Factor 7: Community organisation (average factor loading = 0.665; average structure coefficient = 0.697; average factor loading on refined CFA = 0.872)					
The community−based associations, such as the Edir, in this community are very active.	COMACTCG	−0.874	−0.883	0.915 *	0.918 *
The leaders of community−based associations, like Edir leaders, respond to this community’s concerns.	ACTLDR1	−0.803	−0.840	0.924 *	0.919 *
People in this community get to choose the leaders of their own community−based associations, such as the Edir leaders.	CHOCGLDR	−0.729	−0.802	0.785 *	0.780 *
In this community, people prioritise their own family’s welfare over community development.	OWNWELF	−0.569	−0.575	0.155 *	-
Some households in this community are restricted from community services, such as bed net distribution.	RESTRSER	0.352	0.384	−0.260 *	-

Notes: *Matrix*: Polychoric correlations; *Estimation method*: WLSMV with sandwich estimator to adjust for non-independence of observations within 50 *kebele* clusters; *Extraction*: Combination of Kaiser-Guttman rule (i.e., eigenvalue > 1.0), scree test, goodness-of-fit indices, and substantive justification grounded in theoretical and empirical evidence; *Rotation*: Promax; * *p* ≤ 0.05; ^†^ Refined CFA reflects *post hoc* model adjustments, such as item reduction due to non-salient (loadings < 0.32) or non-significant (two-tailed *p* > 0.05) factor loadings.

**Table 4:** Factor loadings for random split-half samples for EFA and CFA of EFA-derived factor solutions, women.

Factors and Associated Items	Item	Final EFA—Factor Pattern Coefficients (n_W1_ = 366)	Final EFA—Factor Structure Coefficients (n_W1_ = 366)	Baseline CFA (n_W2_ = 360)	Refined ^†^ CFA (n_W2_ = 360)
Factor 1: Social networks and reciprocity (average factor loading = 0.650; average structure coefficient = 0.725; average factor loading on refined CFA = 0.703)					
In this community, I have friends with whom I can share my problems.	HAVEFRND	0.986	0.851	0.863 *	0.863 *
My neighbours sometimes come to me to share their problems and get help.	COME4HLP	0.921	0.842	0.874 *	0.873 *
If you suddenly need some money, you can borrow from a person or group in your community.	BORMONEY	0.617	0.725	0.688 *	0.689 *
If you and your relatives suddenly had to go away for a day or two, you could count on your neighbours to take care of your children.	NEICAREG	0.755	0.789	0.599 *	0.598 *
This is a close-knit community (i.e., people in this community have close personal relationships with each other).	CLOSE	0.542	0.702	0.167	-
I typically accept advice from others in this community.	ADVICE	0.529	0.641	0.616 *	0.616 *
The people of this community will contribute their own money or labour for community development.	CONTRDEV	0.348	0.580	0.684 *	0.683 *
If someone in this community loses a cow or goat, a neighbour will help look for it.	LOSTCOW	0.498	0.672	0.603 *	0.602 *
Factor 2: Social disorder (average factor loading = 0.573; average structure coefficient = 0.546)					
In this community, conflicts like stealing and fighting often occur.	CRIMECON	0.801	0.740	0.366 *	-
In this community, you have to be careful, otherwise your neighbours may cheat you.	CHEATS	0.532	0.500	0.213 *	-
Differences between people, such as the amount of land they own, often causes problems in this community.	DIFPROBS	0.386	0.397	0.900 *	-
Factor 3: Social attachment and personal agency (average factor loading = 0.690; average structure coefficient = 0.793; average factor loading on refined CFA = 0.793)					
Being a member of this community is part of who I am.	IDENTITY	0.907	0.921	0.866 *	0.866 *
I feel proud to be part of this community.	PROUD	0.828	0.906	0.837 *	0.836 *
I feel attached to this community and its people.	ATTACH	0.767	0.850	0.900 *	0.899 *
People in this community accept me as a member of the community.	ACCEPT	0.683	0.806	0.866 *	0.866 *
I have the capacity to achieve my future aims.	SELFEFF	0.521	0.667	0.610 *	0.616 *
I have the ability to contribute to this community’s development.	SEDEV	0.436	0.607	0.674 *	0.676 *
Factor 4: Social response (average factor loading = 0.526; average structure coefficient = 0.639; average factor loading on refined CFA = 0.656)					
Most people in this community have similar beliefs about what is right and what is wrong.	SIMBLIEF	0.793	0.594	0.309 *	-
If the people of this community see crime-like activities, they will do something about it.	INTERCRI	0.586	0.579	0.410 *	0.403 *
People in this community can be trusted.	COMTRUST	0.583	0.701	0.744 *	0.736 *
When there is a problem in this community, people come together to discuss how it should be solved.	COMPRSLV	0.493	0.769	0.861 *	0.851 *
People in this community live in harmony with each other most of the time.	HARMONY	0.462	0.587	0.532 *	0.524 *
If there is a big dispute between two persons, other people from the community will help in solving the problem.	SLVDISPU	0.461	0.642	0.642 *	0.632 *
This is a close-knit community (i.e., people in this community have close personal relationships with each other).	CLOSE	0.443	0.678	0.660 *	0.810 *
If there is a problem that affects the entire community, for instance, crop disease, people in this community will help each other.	HLPCRPDZ	0.385	0.562	0.643 *	0.634 *
Factor 5: Associational participation [in community structures] (average factor loading = 0.784; average structure coefficient = 0.795; average factor loading on refined CFA = 0.802)					
I participate in activities held by any government or NGO-initiated community development group, such as the Development Army.	ACTEXOGP	0.870	0.826	0.809 *	0.808 *
I attend meetings of a community-based association, such as the Edir.	ACTCBGP	0.847	0.854	0.761 *	0.761 *
I participate in activities held by any community-based associations, such as the Edir.	PARTCBGP	0.636	0.704	0.835 *	0.836 *
Factor 6: Common vision (average factor loading = 0.643; average structure coefficient = 0.738; average factor loading on refined CFA = 0.720)					
Most people in this community have similar hopes about the future development of the community.	SIMHOPES	0.898	0.882	0.753 *	0.753 *
People in this community share the same ideas on how village matters should be managed.	COMMGMT	0.718	0.821	0.818 *	0.817 *
Most people in this community have common values, for example, they value hard work.	COMMVALU	0.636	0.729	0.738 *	0.740 *
People in this community have the capacity to make positive changes by coming together.	COLLEFF	0.542	0.730	0.765 *	0.765 *
During a crisis situation, such as a drought, government services are distributed equally by the community to all households in need.	DISTCRIS	0.422	0.526	0.527 *	0.526 *
Factor 7: Community organisation and leadership (average factor loading = 0.649; average structure coefficient = 0.768; average factor loading on refined CFA = 0.777)					
The leaders of community-based associations, like Edir leaders, respond to this community’s concerns.	ACTLDR1	0.919	0.918	0.835 *	0.835 *
The community-based associations, such as the Edir, in this community is very active.	COMACTCG	0.821	0.802	0.774 *	0.773 *
Formal administrative leaders, like the *kebele* manager, provide support to this community.	ACTLDR2	0.549	0.710	0.668 *	0.668 *
People in this community get to choose the leaders of their own community-based associations, such as the Edir leaders.	CHOCGLDR	0.469	0.685	0.822 *	0.822 *
There are people in this community who show strong leadership.	UNOFLDRS	0.489	0.724	0.788 *	0.788 *

Notes: *Matrix*: Polychoric correlations; *Estimation method*: WLSMV with sandwich estimator to adjust for non-independence of observations within 50 *kebele* clusters; *Extraction*: Combination of Kaiser-Guttman rule (i.e., eigenvalue > 1.0), scree test, goodness-of-fit indices, and substantive justification grounded in theoretical and empirical evidence; *Rotation*: Promax * *p* ≤ 0.05; ^†^ Refined CFA reflects *post hoc* model adjustments, such as item reduction due to non-salient (loadings < 0.32) or non-significant (two-tailed *p* > 0.05) factor loadings.

**Table 5:** Final collective efficacy scales, and comparison of single-group (men vs. women) CE factors structures.

Factor *	Item	Survey Item (i.e., Indicator Prompt)	Facets Tapped	Scale
Social response	CLOSE	This is a close-knit community (i.e., people in this community have close personal relationships with each other).	Strength of social bonds within collective/community	P, M, W
	COMPRSLV	When there is a problem in this community, people come together to discuss how it should be solved.	Group problem-solving, conflict-resolution	P, M, W
	COMTRUST	People in this community can be trusted.	Trust in collective/community members	P, M, W
	HLPCRPDZ	If there is a problem that affects the entire community, for instance, crop disease, people in this community will help each other.	Propensity to address community-wide issues, conflict-resolution	P, M, W
	SLVDISPU	If there is a big dispute between two persons, other people from the community will help in solving the problem.	Propensity to address sub-community issues, conflict-resolution	P, M, W
	*HARMONY*	*People in this community live in harmony with each other most of the time.*	*Sense of harmony within the collective/community*	*M*, *W*^∥,¶^
	*SIMBLIEF*	*Most people in this community have similar beliefs about what if right and what is wrong.*	*Collective morals*	*M*
	*SHAREKNO*	*People in this community share knowledge with their neighbour if they learn something new.*	*Information sharing, diffusion of knowledge in collective*	*M*
	*INTERCRI*	*If the people of this community see crime-like activities, they will do something about it.*	*Willingness to intervene*	*W*
Social networks and personal agency	COME4HLP ^†^	My neighbours sometimes come to me to share their problems and get help.	Reciprocity of individual-level problem-solving	P, M, W
	BORMONEY	If you suddenly need some money, you can borrow from a person or group in your community.	Responsiveness of social networks, expectations that help will be given/received by others when in need, cooperating to support one another for one-sided or mutual gain ^§^	P, M, W
	NEICAREG	If you and your relatives suddenly had to go away for a day or two, you could count on your neighbours to take care of your children.		P, M, W
	SEDEV ^†^	I have the ability to contribute to this community’s development.	Individual-level behavioural control over contribution to collective/group goal attainment	P, M, W ^∥^
	SELFEFF ^†^	I have the capacity to achieve my future aims.	Individual behavioural control of personal goal attainment	P, M, W ^∥^
	*HAVEFRND*	*In this community, I have friends with whom I can share my problems.*	*Availability of support networks for individual-level problem solving*	*W*
	*ADVICE*	*I typically accept advice from others in this community.*	*Willingness to receive, access to guidance from others*	*W*
	*CONTRDEV*	*The people of this community will contribute their own money or labour for community development.*	*Common moral principles and codes of behaviour*	*W*
	*LOSTCOW*	*If someone in this community loses a cow or goat, a neighbour will help look for it.*	*Responsiveness of social networks, expectations help will be received, individuals cooperating to support each other*	*W*
Community organisation and leadership	ACTLDR1 ^‡^	The leaders of community-based associations, like Edir leaders, respond to this community’s concerns.	Responsiveness, strength of leaders of endogenous community structures to community concerns	P, M, W
	COMACTCG ^‡^	The community-based associations, such as the Edir, in this community is very active.	Activity level of endogenous community structures	P, M, W
	CHOCGLDR ^‡^	People in this community get to choose the leaders of their own community-based associations, such as the Edir leaders.	Selected representation, civic engagement in endogenous structures	P, M, W
	UNOFLDRS	There are people in this community who show strong leadership.	Presence of individuals demonstrating leadership	P, W, M ^∥^
	ACTLDR2 ^§^	Formal administrative leaders, like the *kebele* manager, provide support to this community.	Supportive leaders of exogenous community structures	P, W, M ^∥^
	*TRUSTLDR*	*This community’s leaders can be trusted.*	*Social trust in community leaders.*	*M*
Associational participation	ACTCBGP ^†,‡^	I attend meetings of a community-based association, such as the Edir.	Personal membership/participation, endogenous community structures	P, M, W
	PARTCBGP ^†,‡^	I participate in activities held by any community-based associations, such as the Edir.	Personal involvement/participation in endogenous group activities	P, M, W
	ACTEXOGP ^†,§^	I attend the meetings of any government or NGO-initiated community development group, such as the Development Army.	Personal membership/participation, exogenous community structures	P, M, W
Social attachment	ACCEPT	People in this community accept me as a member of the community.	Social acceptance within the collective/community	P, M, W
	IDENTITY ^†^	Being a member of this community is part of who I am.	Place identity, sense of belonging	P, M, W
	ATTACH ^†^	I feel attached to this community and its people.	Place attachment	P, M, W
	*PROUD*	*I feel proud to be part of this community.*	*Pride in being a member of the collective/community*	*W*
	COMMGMT	People in this community share the same ideas on how village matters should be managed.	Collective ideals, common civic culture	P, M, W
	SIMHOPES	Most people in this community have similar hopes about the future development of the community.	Common hopes for community goal attainment	P, M, W
Common vision	COMMVALU	Most people in this community have common values, for example, they value hard work.	Shared values, ethics	P, M, W
	COLLEFF	People in this community have the capacity to make positive changes by coming together.	Collective behavioural control; capacity, autonomy	P, M, W
	DISTCRIS	During crisis situations, such as drought, government services are distributed equally by the community to all households in need.	Equal distribution of exogenous resources during crises	P, M, W
	*SHOULDEV*	*People in this community should work together to develop the community.*	*Normative expectations regarding collective action*	*M*

Notes: M = men’s CE scale, W = women’s CE scale, P = Parsimonious CE scale. Items in italicised font appeared in only one gender-specific scale—this meant the item was either absent from one gender-specific scale, or it tapped to a different factor and was re-organised for the purposes of generating a parsimonious scale. Factor labels reflect those from the parsimonious CE scale, and differ slightly in the women’s and men’s CE scales. * Factor titles reflect CE factors in the parsimonious model; ^†^ Self-referent item prompts about the respondent’s own sense of self, agency, autonomy, and level of engagement within his/her community—all other items reflect group-referent items prompts about interpersonal and ecological aspects of the respondent’s community; ^‡^ Items that refer to endogenous community structures (e.g., community-initiated associations)—local endogenous groups used as examples, but should be adapted to the given local context; ^§^ Items that refer to exogenous community structures (e.g., government, NGO-initiated community associations)—local exogenous groups used as examples, but should be adapted; ^∥^ Items re-organised from gender-specific models to produce a parsimonious framework—reflects one gender-specific model; ^¶^ Demonstrated DIF, dropped from final parsimonious scale.

**Table 6:** Factor loadings and fit indices for multiple-group CFA, baseline and final MIMIC, and final CFA (with MIMIC refinement) models.

Factors and Associated Items	Item	Multiple-Group Men (n_EM2_ = 550)	Multiple-Group Women (n_EW2_ = 360)	Baseline MIMIC Model (n_E2_ = 907) ^†^	Final MIMIC Model ^‡^ (n_E2_ = 907) ^†^	Final CFA Model ^§^ (n_E2_ = 907) ^†^
Factor 1: Social response (average baseline MIMIC model factor loading = 0.673; average final MIMIC model factor loading = 0.671; average final CFA model with MIMIC deletions = 0.708)						
This is a close-knit community (i.e., people in this community have close personal relationships with each other).	CLOSE	0.776	0.803	0.782 *	0.783 *	0.773 *
When there is a problem in this community, people come together to discuss how it should be solved.	COMPRSLV	0.695	0.847	0.774 *	0.774 *	0.771 *
If there is a problem that affects the entire community, for instance, crop disease, people in this community will help each other.	HLPCRPDZ	0.712	0.638	0.680 *	0.680 *	0.674 *
People in this community can be trusted.	COMTRUST	0.688	0.727	0.703 *	0.703 *	0.691 *
If there is a big dispute between two persons, other people from the community will help in solving the problem.	SLVDISPU	0.649	0.624	0.635 *	0.635 *	0.629 *
People in this community live in harmony with each other most of the time.	HARMONY	0.422	0.531	0.462 *	0.453 *	-
Factor 2: Social networks and personal agency (average baseline MIMIC model factor loading = 0.663; average final MIMIC model factor loading = 0.663; average final CFA model = 0.663)						
If you suddenly need some money, you can borrow from a person or group in your community.	BORMONEY	0.740	0.667	0.724 *	0.724 *	0.724 *
My neighbours sometimes come to me to share their problems and get help.	COME4HLP	0.758	0.748	0.755 *	0.755 *	0.754 *
If you and your relatives suddenly had to go away for a day or two, you could count on your neighbours to take care of your children.	NEICAREG	0.622	0.562	0.607 *	0.607 *	0.607 *
I have the capacity to achieve my future aims.	SELFEFF	0.533	0.555	0.554 *	0.554 *	0.555 *
I have the ability to contribute to this community’s development.	SEDEV	0.700	0.654	0.676 *	0.676 *	0.677 *
Factor 3: Social attachment (average baseline MIMIC model factor loading = 0.885; average final MIMIC model factor loading = 0.885; average final CFA model = 0.884)						
I feel attached to this community and its people.	ATTACH	0.850	0.915	0.872 *	0.872 *	0.871 *
People in this community accept me as a member of the community.	ACCEPT	0.864	0.879	0.892 *	0.892 *	0.894 *
Being a member of this community is part of who I am.	IDENTITY	0.880	0.835	0.890 *	0.890 *	0.888 *
Factor 4: Common vision (average baseline MIMIC model factor loading = 0.737; average final MIMIC model factor loading = 0.737; average final CFA model = 0.737)						
People in this community share the same ideas on how village matters should be managed.	COMMGMT	0.887	0.815	0.854 *	0.854 *	0.854 *
Most people in this community have similar hopes about the future development of the community.	SIMHOPES	0.834	0.766	0.811 *	0.811 *	0.813 *
Most people in this community have common values, for example, they value hard work.	COMMVALU	0.808	0.743	0.784 *	0.784 *	0.784 *
People in this community have the capacity to make positive changes by coming together.	COLLEFF	0.741	0.760	0.755 *	0.755 *	0.756 *
During crisis situations, such as a drought, government services are distributed equally by the community to all households in need.	DISTCRIS	0.462	0.510	0.481 *	0.481 *	0.478 *
Factor 5: Associational participation (average baseline MIMIC model factor loading = 0.874; average final MIMIC model factor loading = 0.874; average final CFA model = 0.874)						
I attend meetings of a community-based association, such as the Edir.	ACTCBGP	0.979	0.769	0.886 *	0.886 *	0.887 *
I participate in activities held by any community-based associations, such as the Edir.	PARTCBGP	0.933	0.831	0.912 *	0.912 *	0.911 *
I participate in activities held by any government or NGO-initiated community development group, such as the Development Army.	ACTEXOGP	0.830	0.803	0.825 *	0.825 *	0.825 *
Factor 6: Community organisation and leadership (average baseline MIMIC model factor loading = 0.766; average final MIMIC model factor loading = 0.766)						
The leaders of community-based associations, like Edir leaders, respond to this community’s concerns.	ACTLDR1	0.806	0.846	0.829 *	0.829 *	0.829 *
The community-based associations, such as the Edir, in this community are very active.	COMACTCG	0.813	0.778	0.812 *	0.812 *	0.813 *
People in this community get to choose the leaders of their own community-based associations, such as Edir leaders.	CHOCGLDR	0.692	0.810	0.750 *	0.750 *	0.752 *
Formal administrative leaders, like the *kebele* manager, provide support to this community.	ACTLDR2	0.652	0.672	0.669 *	0.669 *	0.667 *
There are people in this community who show strong leadership.	UNOFLDRS	0.762	0.779	0.769 *	0.769 *	0.768 *
**Model fit statistics**						
χ^2^ (df)		1197 (714)		746 (351)	731 (350)	710 (324)
χ^2^ contribution from each group (for multiple group CFA)		598.570	598.907	N/A	N/A	N/A
χ^2^:df		1.676		2.124	2.090	2.191
RMSEA (90% CI)		0.039 (0.035–0.042)		0.035 (0.032–0.039)	0.035 (0.031–0.038)	0.036 (0.033–0.040)
CFI		0.963		0.965	0.966	0.966
TLI		0.964		0.960	0.961	0.960

Notes: *Matrix*: Polychoric correlations; *Estimation method*: WLSMV with sandwich estimator to adjust for non-independence of observations within 50 *kebele* clusters * two-tailed *p* ≤ 0.05; ^†^ Three observations excluded from the MIMIC model due to missing covariate data; ^‡^ Final MIMIC model reflects refined, parsimonious CE measurement model with latent variables regressed on gender and household leadership status plus the inclusion of a direct path between HARMONY and gender; § Final CFA model reflects refined, parsimonious CE measurement model with HARMONY deleted due to DIF.

## Supplementary Materials

The following are available online at http://www.mdpi.com/1660-4601/15/10/2139/s1, Table S1: Univariate descriptive statistics: Frequency of responses by split-halves and gender.

## Appendix A

**Table A1:** Administered 50-Item Collective Efficacy Tool.

Hypothesised Domain	Hypothesised Factors	Item Name	Survey Item (i.e., Prompt)	Description of Hypothesised Facets
Informal social control	Social disorder	HARMONY	People in this community live in harmony with each other most of the time.	Sense of harmony within the collective/community
		*CHEATS*	*In this community, you have to be careful, otherwise your neighbours may cheat you.*	Perceived presence of deceitful individuals
		*CRIMECON*	*In this community, conflicts like stealing and fighting often occur.*	Perceived presence of incivility, delinquent behaviour
		SAFEATHO *	When I am at home alone, I feel safe from threats of crime.	Feeling of safety while at home, in the community
	Social response	SIMBLIEF	Most people in this community have similar beliefs about what is right and what is wrong.	Collective morals
		INTERCRI	If the people of this community see crime-like activities, they will do something about it.	Willingness to intervene
		SLVDISPU	If there is a big dispute between two persons, other people from the community will help in solving the problem.	Community’s propensity to address sub-community-level issues, engage in conflict-resolution
		HLPCRPDZ	If there is a problem that affects the entire community, for instance, crop disease, people in this community will help each other.	Community’s propensity to address community-wide issues, engage in conflict-resolution
		SUPMOURN	If someone in this community had a death in their family, the community will come together to support them while they mourn.	Social support & comforting
		COMPRSLV	When there is a problem in this community, people come together to discuss how it should be solved.	Group problem-solving, conflict-resolution
		CONTRDEV	The people of this community will contribute their own money or labour for community development.	Common moral principles & codes of behaviour
		*DIFPROBS*	*Differences between people, such as the amount of land they own, often causes problems in this community.*	Tolerance
		HAPPYNEI *	I feel happy for my neighbour if they have a good harvest.	Vicarious affective feelings—happiness
Social cohesion	Social capital	COMTRUST	People in this community can be trusted.	Perceived trust in collective/community members
		ADVICE *	I typically accept advice from others in this community.	Willingness to receive, access to guidance from endogenous entities/individuals
		SHAREKNO	People in the community share new knowledge with their neighbour if they learn something new.	Information sharing, diffusion of knowledge within the collective
		CLOSE	This is a close-knit community (i.e., people in this community have close personal relationships with each other).	Strength of social bonds within collective/community
		*OWNWELF*	*In this community, people prioritise their own family’s welfare over community development.*	Commitment to collective development, goal attainment
		LOSTCOW	If someone in this community loses a cow or goat, a neighbour will help look for it.	Perceived responsiveness of social networks, expectations that help will be given to/received by others when in need, individuals cooperating to support one another for either one-sided or mutual gain ^§^
		BORMONEY	If you suddenly need some money, you can borrow from a person or group in your community.	
		NEICAREG	If you & your relatives suddenly had to go away for a day or two, you could count on your neighbours to take care of your children.	
		HAVEFRND *	In this community, I have friends with whom I can share my problems.	Availability of support networks for individual-level problem-solving
		COME4HLP *	My neighbours sometimes come to me to share their problems and get help.	Reciprocity of individual-level problem-solving
		UNOFLDRS	There are people in this community who show strong leadership.	Perceived presence of individuals demonstrating attributes of leadership
		COMACTCG ^†^	The community-based associations, such as the Edir, in this community is very active.	Activity level of endogenous community structures
		ACTLDR1 ^†^	The leaders of community-based associations, like Edir leaders, respond to this community’s concerns.	Responsiveness, strength of the leaders of endogenous community structures to community concerns
		ACTLDR2 ^‡^	Formal administrative leaders, like the *kebele* manager, provide support to this community.	Supportiveness of the leaders of exogenous community structures
		TRUSTLDR	This community’s leaders can be trusted.	Perceived social trust in community leaders
		CHOCGLDR ^†^	People in this community get to choose the leaders of their own community-based associations, such as the Edir leaders.	Civic engagement in endogenous community structures, community-selected representation
		COPARTCG ^†,‡^	Most people in this community participate in community associations.	Community engagement in endogenous and exogenous community structures
		ACTCBGP *^,†^	I attend meetings of a community-based association, such as the Edir.	Personal associational membership/participation, endogenous community structures
		PARTCBGP *^,†^	I participate in activities held by any community-based associations, such as the Edir.	Personal involvement/participation in activities organised by endogenous community structures
		ACTEXOGP *^,‡^	I attend the meetings of any government or NGO-initiated community development group, such as the Development Army.	Personal associational membership/participation, exogenous community structures
		PAREXOGP *^,‡^	I participate in activities held by any government or NGO-initiated community development group, such as the Development Army.	Personal involvement/participation in activities organised by exogenous community structures
	Social equity	COMMGDEC	When community groups make decisions, they are pleasing and good for most of the households in this community.	Social equity prioritised during community decision-making processes
		*BRIBELDR*	*Sometimes people need to bribe community leaders in order to get things done.*	Corruption among community leaders
		DISTCRIS	During a crisis situation, such as drought, government services are distributed equally by the community to all households in need.	Equal distribution of exogenous resources during crises
		*RESTRSER*	*Some households in this community are restricted from community services, such as bed net distribution.*	*Social injustice, restrictions to resources*
	Common values	COMMVALU	Most people in this community have common values, for example, they value hard work.	Shared values, ethics
		SIMHOPES	Most people in this community have similar hopes about the future development of the community.	Common hopes for community goal attainment, performance
		COMMGMT	People in this community share the same ideas on how village matters should be managed.	Collective ideals, common civic culture
	Community attachment	ACCEPT	People in this community accept me as a member of the community.	Social acceptance within the collective/community
		ATTACH *	I feel attached to this community and its people.	Place attachment
		PROUD *	I feel proud to be part of this community.	Pride in being a member of the collective/community
		IDENTITY *	Being a member of this community is part of who I am.	Place identity, sense of belonging
Behavioural control	Agency	SELFEFF *	I have the capacity to achieve my future aims.	Perceived individual-level behavioural control over personal goal attainment
		SEDEV *	I have the ability to contribute to this community’s development.	Perceived individual-level behavioural control over contribution to collective/group goal attainment
		COLLEFF	People in this community have the capacity to make positive changes by coming together.	Perceived community-level behavioural control; capacity and autonomy control beliefs
		*EXOASSIS*	*This community needs assistance from others outside the community in order to make positive changes.*	Perceived reliance on exogenous support to facilitate goal attainment
		SHOULDEV	People in this community should work together to develop the community.	Normative expectations regarding collective action

Notes: Indicated sub-constructs reflect those conceptualised via our hypothesised collective efficacy framework. Items presented in italicised font were hypothesised to have an inverse relationship with CE. Given the various conceptualisations of these latent constructs, substantive justification existed for the re-conceptualisation articulated in our EFA-derived factor structures. * self-referent item prompts about the respondent’s own sense of self, agency, autonomy, and level of engagement within his/her community; all other items reflect group-referent items prompts about ecological aspects of the respondent’s community; ^†^ items that refer to endogenous community structures (e.g., community-initiated/organised community associations)—local endogenous structures used as examples, but should be adapted to the given local context; ^‡^ items that refer to exogenous community structures (e.g., government or NGO-initiated/organised community associations)—local exogenous structures used as examples, but should be adapted to the given context; ^§^ measured through different scenarios reflecting different levels of urgency/need.

**Table A2:** Factor Loadings from Preliminary CFA of Our Hypothesised CE Framework, Men.

Factors and Associated Items	Item	Initial Prelim. CFA (n_M_ = 1105)	Refined ^†^ Prelim. CFA (n_M_ = 1105)
Factor 1: Agency (average factor loading on refined CFA = 0.735)			
People in this community have the capacity to make positive changes by coming together.	COLLEFF	0.804 *	0.799 *
I have the ability to contribute to this community’s development.	SEDEV	0.775 *	0.778 *
People in this community should work together to develop the community.	SHOULDEV	0.747 *	0.744 *
I have the capacity to achieve my future aims.	SELFEFF	0.612 *	0.617 *
This community needs assistance from others outside the community in order to make positive changes.	EXOASSIS	0.011	-
Factor 2: Common values (average factor loading on refined CFA = 0.831)			
Most people in this community have common values, for example, they value hard work.	COMMVALU	0.779 *	0.778 *
People in this community share the same ideas on how village matters should be managed.	COMMGMT	0.877 *	0.877 *
Most people in this community have similar hopes about the future development of the community.	SIMHOPES	0.838 *	0.839 *
Factor 3: Social response (average factor loading on refined CFA = 0.573)			
If the people of this community see crime-like activities, they will do something about it.	INTERCRI	0.446 *	0.444 *
When there is a problem in this community, people come together to discuss how it should be solved.	COMPRSLV	0.718 *	0.719 *
If there is a big dispute between two persons, other people from the community will help in solving the problem.	SLVDISPU	0.710 *	0.712 *
If there is a problem that affects the entire community, for instance, crop disease, people in this community will help each other.	HLPCRPDZ	0.689 *	0.686 *
The people of this community will contribute their own money or labour for community development.	CONTRDEV	0.660 *	0.660 *
If someone in this community had a death in their family, the community will come together to support them while they mourn.	SUPMOURN	0.520 *	0.521 *
I feel happy for my neighbour if they have a good harvest.	HAPPYNEI	0.445 *	0.442 *
Most people in this community have similar beliefs about what is right and what is wrong.	SIMBLIEF	0.399 *	0.399 *
Differences between people, such as the amount of land they own, often causes problems in this community.	DIFPROBS	−0.267 *	-
Factor 4: Social order (average factor loading on refined CFA = 0.539)			
People in this community live in harmony with each other most of the time.	HARMONY	0.818 *	0.984 *
In this community, you have to be careful, otherwise your neighbours may cheat you.	CHEATS	−0.304 *	−0.305 *
In this community, conflicts like stealing and fighting often occur.	CRIMECON	−0.284 *	−0.327 *
When I am at home alone, I feel safe from threats of crime.	SAFEATHO	0.150 *	-
Factor 5: Social capital (average factor loading on refined CFA = 0.671)			
People in this community can be trusted.	COMTRUST	0.631 *	0.631 *
I typically accept advice from others in this community.	ADVICE	0.591 *	0.593 *
People in the community share new knowledge with their neighbour if they learn something new.	SHAREKNO	0.646 *	0.648 *
This is a close-knit community (i.e., people in this community have close personal relationships with each other).	CLOSE	0.677 *	0.676 *
If someone in this community loses a cow or goat, a neighbour will help look for it.	LOSTCOW	0.535 *	0.533 *
If you suddenly need some money, you can borrow from a person or group in your community.	BORMONEY	0.605 *	0.609 *
If you and your relatives suddenly had to go away for a day or two, you could count on your neighbours to take care of your children.	NEICAREG	0.588 *	0.588 *
There are people in this community who show strong leadership.	UNOFLDRS	0.660 *	0.656 *
The community-based associations, such as the Edir, in this community are very active.	COMACTCG	0.622 *	0.626 *
The leaders of community-based associations, like Edir leaders, respond to this community’s concerns.	ACTLDR1	0.615 *	0.614 *
Formal administrative leaders, like the *kebele* manager, provide support to this community.	ACTLDR2	0.619 *	0.610 *
This community’s leaders can be trusted.	TRUSTLDR	0.766 *	0.764 *
People in this community get to choose the leaders of their own community-based associations, such as the Edir leaders.	CHOCGLDR	0.521 *	0.520 *
In this community, I have friends with whom I can share my problems.	HAVEFRND	-	-
My neighbours sometimes come to me to share their problems and get help.	COME4HLP	0.633 *	0.637 *
Most people in this community participate in community associations.	COPARTCG	0.700 *	0.698 *
I attend meetings of a community-based association, such as the Edir.	ACTCBGP	0.881 *	0.885 *
I participate in activities held by any community-based associations, such as the Edir.	PARTCBGP	0.888 *	0.892 *
I participate in activities held by any government or NGO-initiated community development group, such as the Development Army.	ACTEXOGP	0.804 *	0.804 *
I participate in activities held by any government or NGO-initiated community development group, such as the Development Army.	PAREXOGP	0.781 *	0.781 *
In this community, people prioritise their own family’s welfare over community development.	OWNWELF	0.139 *	-
Factor 6: Social equity (average factor loading on refined CFA = 0.573)			
When community groups make decisions, they are pleasing and good for most of the households in this community.	COMMGDEC	0.728 *	-
During a crisis situation, such as a drought, government services are distributed equally by the community to all households in need.	DISTCRIS	0.435 *	-
Sometimes people need to bribe community leaders in order to get things done.	BRIBELDR	−0.226 *	-
Some households in this community are restricted from community services, such as bed net distribution.	RESTRSER	−0.223 *	-
Factor 7: Community attachment (average factor loading on refined CFA = 0.865)			
I feel attached to this community and its people.	ATTACH	0.813 *	0.814 *
I feel proud to be part of this community.	PROUD	0.936 *	0.935 *
Being a member of this community is part of who I am.	IDENTITY	0.868 *	0.869 *
People in this community accept me as a member of the community.	ACCEPT	0.842 *	0.841 *

Notes: *Matrix*: Polychoric correlations; *Estimation method*: WLSMV with sandwich estimator to adjust for non-independence of observations within 50 *kebele* clusters; *Extraction*: Combination of Kaiser-Guttman rule (i.e., eigenvalue > 1.0), scree test, goodness-of-fit indices, and substantive justification grounded in theoretical and empirical evidence; *Rotation*: Promax; * two-tailed *p* ≤ 0.05; ^†^ Refined CFA reflects *post hoc* model adjustments, such as item reduction due to non-salient (loadings < 0.32) or non-significant (two-tailed *p* > 0.05) factor loadings.

**Table A3:** Factor Loadings from Preliminary CFA of Our Hypothesised CE Framework, Women.

Factors and Associated Items	Item	Initial Prelim. CFA (n_W_ = 726)	Refined ^†^ Prelim. CFA (n_W_ = 726)
Factor 1: Agency (average factor loading on refined CFA = 0.723)			
People in this community have the capacity to make positive changes by coming together.	COLLEFF	0.812 *	0.807 *
I have the ability to contribute to this community’s development.	SEDEV	0.703 *	0.703 *
People in this community should work together to develop the community.	SHOULDEV	0.692 *	0.689 *
I have the capacity to achieve my future aims.	SELFEFF	0.691 *	0.692 *
This community needs assistance from others outside the community in order to make positive changes.	EXOASSIS	0.231 *	-
Factor 2: Common value (average factor loading on refined CFA = 0.811)			
Most people in this community have common values, for example, they value hard work.	COMMVALU	0.763 *	0.762 *
People in this community share the same ideas on how village matters should be managed.	COMMGMT	0.874 *	0.878 *
Most people in this community have similar hopes about the future development of the community.	SIMHOPES	0.794 *	0.792 *
Factor 3: Social response (average factor loading on refined CFA = 0.623)			
If the people of this community see crime-like activities, they will do something about it.	INTERCRI	0.474 *	0.475 *
When there is a problem in this community, people come together to discuss how it should be solved.	COMPRSLV	0.888 *	0.888 *
If someone in this community had a death in their family, the community will come together to support them while they mourn.	SUPMOURN	0.732 *	0.740 *
The people of this community will contribute their own money or labour for community development.	CONTRDEV	0.699 *	0.700 *
If there is a big dispute between two persons, other people from the community will help in solving the problem.	SLVDISPU	0.681 *	0.679 *
If there is a problem that affects the entire community, for instance, crop disease, people in this community will help each other.	HLPCRPDZ	0.659 *	0.659 *
I feel happy for my neighbour if they have a good harvest.	HAPPYNEI	0.523 *	0.526 *
Most people in this community have similar beliefs about what is right and what is wrong.	SIMBLIEF	0.331 *	0.316 *
Differences between people, such as the amount of land they own, often causes problems in this community.	DIFPROBS	0.009	-
Factor 4: Social order (dropped from factor model given only 1 item with salient factor loading)			
People in this community live in harmony with each other most of the time.	HARMONY	0.666 *	-
In this community, you have to be careful, otherwise your neighbours may cheat you.	CHEATS	0.053	-
In this community, conflicts like stealing and fighting often occur.	CRIMECON	−0.040	-
When I am at home alone, I feel safe from threats of crime.	SAFEATHO	0.242 *	-
Factor 5: Social capital (average factor loading on refined CFA = 0.661)			
People in this community can be trusted.	COMTRUST	0.644 *	0.636 *
I typically accept advice from others in this community.	ADVICE	0.601 *	0.605 *
People in the community share new knowledge with their neighbour if they learn something new.	SHAREKNO	0.630 *	0.628 *
This is a close-knit community (i.e., people in this community have close personal relationships with each other).	CLOSE	0.749 *	0.744 *
In this community, people prioritise their own family’s welfare over community development.	OWNWELF	0.335 *	0.342 *
If someone in this community loses a cow or goat, a neighbour will help look for it.	LOSTCOW	0.580 *	0.585 *
If you suddenly need some money, you can borrow from a person or group in your community.	BORMONEY	0.636 *	0.635 *
If you and your relatives suddenly had to go away for a day or two, you could count on your neighbours to take care of your children.	NEICAREG	0.602 *	0.602 *
There are people in this community who show strong leadership.	UNOFLDRS	0.726 *	0.723 *
The community-based associations, such as the Edir, in this community are very active.	COMACTCG	0.690 *	0.692 *
The leaders of community-based associations, like Edir leaders, respond to this community’s concerns.	ACTLDR1	0.770 *	0.770 *
Formal administrative leaders, like the kebele manager, provide support to this community.	ACTLDR2	0.660 *	0.656 *
This community’s leaders can be trusted.	TRUSTLDR	0.713 *	0.702 *
People in this community get to choose the leaders of their own community-based associations, such as the Edir leaders.	CHOCGLDR	0.741 *	0.742 *
In this community, I have friends with whom I can share my problems.	HAVEFRND	0.756 *	0.759 *
My neighbours sometimes come to me to share their problems and get help.	COME4HLP	0.768 *	0.772 *
Most people in this community participate in community associations.	COPARTCG	0.624 *	0.624 *
I attend meetings of a community-based association, such as the Edir.	ACTCBGP	0.584 *	0.587 *
I participate in activities held by any community-based associations, such as the Edir.	PARTCBGP	0.579 *	0.580 *
I participate in activities held by any government or NGO-initiated community development group, such as the Development Army.	ACTEXOGP	0.749 *	0.756 *
I participate in activities held by any government or NGO-initiated community development group, such as the Development Army.	PAREXOGP	0.729 *	0.733 *
Factor 6: Social equity (dropped from factor model given only 2 items with salient factor loadings)			
When community groups make decisions, they are pleasing and good for most of the households in this community.	COMMGDEC	0.650 *	-
Sometimes people need to bribe community leaders in order to get things done.	BRIBELDR	−0.227 *	-
During a crisis situation, such as a drought, government services are distributed equally by the community to all households in need.	DISTCRIS	0.509 *	-
Some households in this community are restricted from community services, such as bed net distribution.	RESTRSER	−0.105 *	-
Factor 7: Community attachment (average factor loading on refined CFA = 0.884)			
I feel attached to this community and its people.	ATTACH	0.889 *	0.888 *
Being a member of this community is part of who I am.	IDENTITY	0.882 *	0.882 *
People in this community accept me as a member of the community.	ACCEPT	0.875 *	0.875 *
I feel proud to be part of this community.	PROUD	0.889 *	0.890 *

Notes: *Matrix*: Polychoric correlations; *Estimation method*: WLSMV with sandwich estimator to adjust for non-independence of observations within 50 *kebele* clusters; *Extraction*: Combination of Kaiser-Guttman rule (i.e., eigenvalue > 1.0), scree test, goodness-of-fit indices, and substantive justification grounded in theoretical and empirical evidence; *Rotation*: Promax * two tailed *p* ≤ 0.05; ^†^ Refined CFA reflects post hoc model adjustments, such as item reduction due to non-salient (loadings < 0.32) or non-significant (two-tailed *p* > 0.05) factor loadings.

**Table A4:** Comparison of Model Fit Statistics from Preliminary CFA of Our Hypothesised CE Framework and CFA of EFA-Derived CE Factor Solutions.

Fit Statistic	Preliminary CFA of Hypothesised CE Framework				CFA of EFA-Derived Factor Solutions			
	Men		Women		Men		Women	
	Initial CFA	Refined CFA *	Initial CFA	Refined CFA *	Initial CFA	Refined CFA *	Initial CFA	Refined CFA *
χ^2^	2312.227	1991.259	2810.783	2488.535	721.300	618.517	858.865	755.374
df	1106	764	1154	730	504	413	607	480
χ^2^:df	2.091	2.606	2.436	3.409	1.431	1.498	1.415	1.574
*p*-value	<0.001	<0.001	<0.001	<0.001	<0.001	<0.001	<0.001	<0.001
RMSEA (90% CI)	0.031 (0.030–0.033)	0.038 (0.036–0.040)	0.044 (0.042–0.047)	0.058 (0.055–0.060)	0.028 (0.023–0.032)	0.030 (0.025–0.035)	0.034 (0.029–0.039)	0.040 (0.034–0.045)
CFI	0.911	0.911	0.897	0.895	0.970	0.971	0.964	0.962
TLI	0.905	0.904	0.891	0.888	0.966	0.968	0.960	0.958
WRMR	1.962	2.089	2.124	2.344	1.047	1.005	1.137	1.168

Notes: WLSMV estimation with sandwich estimator to adjust for non-independence of observations with 50 *kebele* clusters; * Refined CFA reflects *post hoc* model adjustments, such as item reduction due to non-salient (loadings < 0.32) or non-significant (two-tailed *p* > 0.05) factor loadings; **Abbreviations:** df = degrees of freedom; RMSEA = Root Mean Square Error of Approximation; CFI = Comparative Fit Index; TLI = Tucker-Lewis Index; WRMR = Weighted Root Mean Square Residual.

**Table A5:** Model Fit Statistics for CFA of Refined Single-Group and Parsimonious (Overlapping Items) Models.

Fit Statistic	Refined, Single-Group CFA Models *		Fit of Male, Female Data to Parsimonious Model	
	Men	Women	Men	Women
	Saturated Model, All Items	Saturated Model, All Items	Overlapping Items Only	Overlapping Items Only
χ^2^	618.517	755.374	536.910	503.073
df	413	480	309	309
χ^2^:df	1.498	1.574	1.738	1.628
*p*-value	<0.001	<0.001	<0.001	<0.001
RMSEA (90% CI)	0.030 (0.025–0.035)	0.040 (0.034–0.045)	0.037 (0.031–0.042)	0.042 (0.035–0.048)
CFI	0.971	0.962	0.965	0.967
TLI	0.968	0.958	0.961	0.963
WRMR	1.005	1.168	1.148	1.070

Notes: WLSMV estimation with sandwich estimator to adjust for non-independence of observations with 50 *kebele* clusters; * Refined CFA models reflect *post hoc* model adjustments, such as item reduction due to non-salient (loadings < 0.32) or non-significant (two-tailed *p* > 0.05) factor loadings; Abbreviations: df = degrees of freedom; RMSEA = Root Mean Square Error of Approximation; CFI = Comparative Fit Index; TLI = Tucker-Lewis Index; WRMR = Weighted Root Mean Square Residual.

**Table A6:** Competing MIMIC Models: Fit Statistics, Unstandardised B Estimates, Standard Errors, and Standardised β Estimates.

Model	*n*	χ^2^ (df)	Δ χ^2^ (df)	RMSEA (90% CI)	CFI	TFI	B	S.E.	β
MIMIC models with gender covariate only									
1. Baseline MIMIC model—i.e., CE measurement model with ALL latent factors regressed on gender, no direct effects between gender & item indicators	910	731 (330)	N/A	0.037 (0.033–0.040)	0.966	0.961	-	-	-
2. Model 1 (i.e., ALL latent factors regressed on gender) + direct path between HARMONY item indicator and gender	910	717 (329)	31 *	0.036 (0.032–0.040)	0.967	0.962	−0.453 *	0.081	−0.444 *
MIMIC models with gender AND household leadership status covariates									
3. More saturated baseline MIMIC model with ALL latent factors regressed on gender AND leadership, no direct effects between covariates & items	907	746 (351)	N/A	0.035 (0.032–0.039)	0.965	0.960	-	-	-
4. Model 3 (ALL latent factors regressed on gender and leadership covariates) + direct path between HARMONY item indicator and gender	907	731 (350)	32 *	0.035 (0.031–0.038)	0.966	0.961	−0.457 *	0.081	−0.448 *
5. Model 4 + direct path between ACTEXOGP item indicator and leadership covariate	907	727 (349)	15 *	0.035 (0.031–0.038)	0.967	0.961	0.321 *	0.083	0.299 *
6. More saturated baseline MIMIC model with ONLY latent factors significant on Model 3 regressed on gender and leadership	907	730 (355)	N/A	0.034 (0.031–0.038)	0.967	0.962	-	-	-

Notes: *Matrix*: Polychoric correlations; *Estimation method*: WLSMV with sandwich estimator to adjust for non-independence of observations within 50 *kebele* clusters; Δ χ^2^ assessed via DIFFTEST; B: unstandardised estimate; S.E. = standard error; β: standardised estimate; * *p* ≤ 0.001, though χ^2^ statistics are sensitive to sample size, therefore, DIFFTEST statistics are likely to be significant with large samples; Baseline MIMIC model regressed latent variables on gender and household leadership status; additional direct effect paths between gender and leadership covariates incorporated in subsequent models via step-wise forward selection based on the magnitude of the item indicator’s modification index; Model 4 reflects final MIMIC model (i.e., model identified with the best fit and most substantively justified factor structure.
