# Supplementary material for: Collective Efficacy: Development and Validation of a Measurement Scale for Use in Public Health and Development Programmes
Source: Int J Environ Res Public Health. 2018 Sep 28;15(10):2139. doi: 10.3390/ijerph15102139 (PMC6211028; doi:10.3390/ijerph15102139)
Supplement: Supplementary file 1 [file ijerph-15-02139-s001.pdf]

International Journal of
Environmental Research and Public Health

MDPI

Supplemental Material

Collective efficacy: Development and validation of a measurement scale for use in public health and development programmes

Maryann G. Delea [1,2,*], Gloria D. Sclar [2], Mulat Woreta [3], Regine Haardörfer [4], Corey L. Nagel [5], Bethany A. Caruso [2], Robert Dreibelbis [1], Abebe G. Gobezayehu [3], Thomas F. Clasen [1,2],

ADDITIONAL METHODS

Additional sampling methodology related to the larger Andilaye trial

For the larger *Andilaye* trial, we employed a structured sampling strategy to randomly select eligible sub-district (*kebele*) clusters and study households. The primary sampling unit for the *Andilaye* trial was the *kebele*; specifically, any rural or peri-urban *kebele* within three districts (*woredas* – Bahir Dar Zuria, Fogera, and Farta) of Amhara, Ethiopia that is accessible throughout the course of the year. The ultimate sampling unit for this study was the household; specifically, any household residing in a targeted, sentinel village (*gott)* within a randomly selected study *kebele*. We randomly selected households for inclusion in the study by using a random number generator to identity approximately 30 households from a sampling frame of all households in the selected *gott* that had at least one child between the ages of 1-9 years. We conducted our CE sub-study amongst the households enrolled in the *Andilaye* trail.

When the primary target respondents for our survey were not present, one of the following adult household members was engaged (listed preferentially): any female household member who serves as a caregiver, any male household member serves as a caregiver, any adult household member.

Additional EFA details

Priority was placed on selecting solutions with model-estimated correlation matrices close in value to the original sample correlation matrices, and residual correlation matrices with values close to zero. Such results indicated that factor solutions with the respective number of retained factors were sufficient for explaining the inter-correlations amongst the observed variables.

Criteria to identify models with simple structure

The following, adapted from Thurstone's criteria [1], were to identify models with simple structure and guide iterative re-analysis of CE measurement models subsequent to item reduction via EFA:

- Each item produces at least one zero loading on some factor.
- Each factor is measured by a set of items with high loadings on the factor, and at least as many zero loadings as there are factors.
- Each item has a high loading on one factor (i.e., primary loading), and a trivial or close to zero loading on all remaining factors.
- Each pair of factors should have a large proportion of zero loadings on both factors (if there are four or more factors total).
- Each pair of factors should have only a few complex variables.

We used established guidance [2] to define a zero loading, which we deemed as any factor loading between -0.100 and +0.100. Our definition of significant loading was informed by empirical evidence as well. Factor loadings with an absolute value greater than 0.320 were considered salient.

Items with factor loadings less than this threshold measured the latent factors poorly, and were eliminated in a step-wise manner [3]. It is worth noting that no broadly accepted guidelines exist for saliency of factor loadings, but pattern coefficients in the range of 0.300-0.400 are often interpreted by analysts as salient in applied research [4]. We defined complex variables as items with factor loadings of |0.300| on more than one factor [4].

Justification of factor extraction approach

Factor retention was not solely based on the Kaiser-Guttman rule (i.e., eigenvalue >1.0 [5]), but also considered heuristic descriptive guides (i.e., scree-plot), goodness-of-fit, and other substantive justification (e.g., results from cognitive interviews, theory and other evidence). The last factor extracted for the men's CE model had an eigenvalue of 1.118; the first factor not retained had an eigenvalue of 1.029. The eigenvalue for the last factor extracted for the women's CE model was 1.336; the first factor not retained had an eigenvalue of 1.068. While the first factor not retained for both men's and women's CE measurement models had values above 1.0 threshold, the retention of those factors was not warranted by strong substantive or statistical justification [4, 6, 7]. Including those factors merely because their eigenvalues were slightly greater than 1.0 would reflect the sole use of a mathematically-based descriptive guide for item retention. Such an approach would go against our pre-analysis plan, disregard heuristic and model fit criteria, and important empirical and theoretical considerations (e.g., results from cognitive interviews, pilot testing of the CE instrument and other prior theoretical and empirical evidence).

In addition, many methodologists have criticised and demonstrated that the Kaiser-Guttman rule can tend to result in overfactoring or underfactoring given sampling error may influence eigenvalues [2, 4, 8]. While identifying and retaining too few factors (i.e., underfactoring) may result in an oversimplified understanding of a construct, retaining too many factors (i.e., overfactoring) may lead to violation of parsimony, which is one primary goal of EFA [4]. Whether over- or underfactoring occurs, the factor solution that results may lead to unreliable factors and/or errors in interpretation [2, 8]. Given the more parsimonious (i.e., eight-factor) measurement models were supported by our knowledge of the existing theoretical and empirical literature base, and other non-mathematically-based criteria, we felt our factor extraction and retention decisions were sufficiently justified.

ADDITIONAL RESULTS

Univariate statistics: CE survey items

Our CE survey included 50 items for factoring (Appendix A). The top five items to which respondents most frequently selected "completely agree" aligned for men and women, though there were some differences with regard to the proportions of those responses between genders (Appendix SA). These items reflected those related to social solidarity or support for one's community members and a sense of pride about being a part of the community: "If someone in this community had a death in their family, the community will come together to support them while they mourn" (94% of men, 91% of women); "I feel happy for my neighbour if they have a good harvest" (96% of men, 92% of women); "I feel proud to be part of this community" (91% of men, 73% of women); "If someone in this community loses a cow or goat, a neighbour will help look for it" (89% of men, 90% of women); "People in this community get to choose the leaders of their own community-based associations, such as Edir leaders" (86% of men, 73% of women). The items to which respondents most frequently selected "completely disagree" also aligned between genders. These items reflected those related to social disorder and inequity: "Sometimes people need to bribe community leaders in order to get things done" (71% of men, 52% of women); "Some households in this community are restricted from community services, such as bed net distribution" (71% of men, 46% of women); "In this community, conflicts like stealing and fighting often occur" (29% of men, 47% of women); "In this community, you have to be careful, otherwise your neighbours will cheat you" (25% of men, 30% of women). In terms of normality of item response distributions, men had 27 items, and women had 15 items with skewness outside of the suggested range (Appendix SA). The WLSMV estimator we employed for

our factor analyses makes no distribution assumptions for observed variables, and only assumes a normal latent distribution underlying each observed categorical variable [9], so no action was taken to address any non-normal item distributions [4].

Interpretation of factor loadings

It is acceptable and appropriate to consider factor loadings that vary in magnitude across the various items tapping to a latent factor, as the magnitude of an item's factor loading reflects the proximity of the relationship between the item and the factor to which it taps [10]. Factor loadings may therefore vary in magnitude across the items tapping to a factor based on the proximity of those relationships [10]. Items that are conceptually less influential (i.e., less proximal) to a given latent factor could demonstrate a lower factor loading without necessarily signaling poor quality of the latent factor and poor validity of the measurement model [11]. An item indicator that almost perfectly reflects a given latent factor should be very highly correlated with it (e.g., as represented by a factor loading in the range of 0.800-0.900). However, other items tapping to the latent factor that are conceptually less important or proximal to the factor can, and theoretically should demonstrate lower factor loadings [10, 12].

Additional preliminary CFA results

We moved forward with *post hoc* model refinements of preliminary CFA models to eliminate non-salient and non-significant factor loadings as well as any factors with insufficient component saturation. For the men's model, this resulted in the elimination of nine items. One item (HAVEFRND) was eliminated because it had less than minimal variance (i.e., a response category with zero observations). Five items were eliminated for non-salient factor loadings (OWNWELF=0.140, SAFEATHO=0.151, RESTRSER=-0.223, BRIBELDR=-0.226, DIFPROBS=-0.267), and one item was eliminated because it had both a non-salient and non-significant loading on its designated factor (EXOASSIS=0.011, p=0.793). After eliminating items that were non-significant and non-salient, one factor (social equity) remained with only two items, which we did not deem sufficient for component saturation. We therefore eliminated that factor and the remaining two items which otherwise demonstrated salient and significant loadings (COMMGDEC=0.717, DISTCRIS=0.428). The standardised estimates of the remaining factor loadings from this model were acceptable (Appendix B), and all remaining factors co-varied significantly. The refined preliminary CFA model of the hypothesized CE framework demonstrated adequate absolute model fit (χ^2:df ratio = 2.606, RSMEA=0.038 [0.036 – 0.040]), but still poor incremental fit (CFI=0.911, TLI=0.904). These results suggest that our hypothesised CE framework represented a plausible structure of the mechanisms through which the CE process operates amongst men in the Ethiopian context. However, poor incremental fit statistics suggested that this may not have been the best fitting model framework.

For the women's model, we eliminated a total of ten items. Four items were eliminated as a result of non-salient factor loadings on the designated factor (RESTRSER=-0.105, BRIBELDR=-0.227, EXOASSIS=0.231, SAFEATHO=0.242). Three items were eliminated due to non-salient and non-significant factor loadings on the designated factor (DIFPROBS=0.009, p=0.868; CRIMECON=-0.040, p=0.543; CHEATS=0.053, p=0.324). Two factors and their three items were eliminated because the factors demonstrated insufficient component saturation (the factor representing social order with its HARMONY item, and the factor representing social equity with its COMMGDEC and DISTCRIS items). The refined preliminary CFA model only marginally reflected the actual hypothesised framework, as two factor loadings were non-salient (social order and social equity). The standardised estimates of the remaining factor loadings from the resulting model were acceptable (Appendix C). Both absolute and incremental fit statistics indicated poor fitness of the resulting women's factor model (χ^2:df ratio=3.409, RSMEA=0.058 [0.055–0.060]; CFI=0.895, TLI=0.888). This means that the data failed to validate the hypothesised CE framework for women respondents, indicating the framework did not reflect the mechanisms through which the CE process operates for women in the rural Ethiopian context.

There was considerable overlap in the items eliminated from both men and women refined, preliminary CFA models. All but one (OWNWELF) of the items eliminated from the men's model were also eliminated from the women's model, and five of the ten items eliminated from the women's model were also eliminated from the men's model (SAFEATHO, RESTRSER, BRIBELDR, DIFPROBS, and EXOASSIS).

Additional EFA and CFA results

Complete EFA results reflect coefficients from both rotated (Promax) pattern and structure matrices along with initial and refined CFA results. While not all factor loadings demonstrated in Tables 3 and 4 are in the range of excellent to very good – though they are still in the acceptable range – we hypothesise that some of those items are conceptually more distal (i.e., marginally less important) to the measurement of the latent factor. We present further details regarding both men and women EFA-derived measurement models in subsequent sub-sections.

Additional details regarding the men's EFA and CFA results

During the EFA analyses, we eliminated three items (HAVEFRND, HAPPYNEI, PROUD) due to less than minimal variance (i.e., no observations in one or more item response category) that prevented the EFA from being processed in MPlus. We also eliminated twelve items, in a step-wise manner: ten items were eliminated because they had no salient loadings on any factor (BRIBELDR, EXOASSIS, SAFEATHO, COPARTCG, CHEATS, INTERCRI, COMMGDEC, CONTRDEV, SUPMOURN, LOSTCOW); one item (PAREXOGP) was eliminated due to evidence of extreme multicollinearity with another related item that loaded to the factor; and one item (CRIMECON) was eliminated because although its pattern coefficient was salient, its structure coefficient was not. This resulted in a 35-item men's CE measurement model (with two complex variables) that tapped to seven factors of CE: social response, social networks and personal agency, social attachment, common vision, community leadership, associational participation, and community organisation.

Factor one, labelled "social response" corresponded to the informal social control domain, though it also tapped to certain aspects of cognitive social capital (e.g., trust in community members, reciprocity of knowledge) that may influence social response. The factor contained nine items that tap to various facets of perceptions regarding the community's propensity to address community- and sub-community level issues, including social disorder (e.g., harmony, problem solving, conflict-resolution, common moral principles and codes of behaviour), support in times of crisis, and tolerance. The concepts reflected in this factor align closely with our hypothesised operational definition of social control, described as an absence of general conflict and threats to the existing order, effective informal social control, tolerance, and intergroup cooperation (Table 1).

Factor two, labelled "social networks & personal agency" corresponded to the cognitive social capital domain, though it also tapped to structural social capital, as it reflects the strength and responsiveness of one's social structures. The factor comprised of five items that relate to issues surrounding supporting networks and individuals cooperating to support one another for either mutual or one-sided gain. Two items related to self-efficacy loaded to this factor. This suggests that for men, one's perspectives regarding personal agency (i.e., individual behavioural control) is linked to perceived expectations that help will be given to or received from others, when needed [13].

Factors three and four corresponded to the social cohesion domain. Factor three, labelled "social attachment" included five items that tap to concepts related to place identity, community acceptance and attachment, and collective agency. Factor four, labelled "common vision" was comprised of six items that reflect shared norms (perceptions of normative expectations regarding contributions to community development) and culture (common values, hopes for the future, ideas about how the community should be managed), social equity (equal distribution of goods in times of crisis), and perceptions regarding community-level agency.

Factors five, six, and seven pertained to the structural social capital domain. Factor five, labelled "community leadership" reflected four items tapping to various aspects of social trust, support, and strength of leadership of formal administrative leaders and both formal and informal community

leaders. Factor six, labelled "associational participation" corresponded to the respondent's personal involvement in established community structures – both exogenously and endogenously organised. The three constituent items reflect both membership (as indicated by meeting attendance) and participation in associational activities. Factor seven, labelled "community organisation" corresponded to various aspects of community organisation, including the activity level of endogenously organised community associations and leaders thereof, community-selected representation, prioritisation of community development, and social justice and equity.

During CFA, we moved forward with *post hoc* model refinements to eliminate non-salient and non-significant factor loadings as well as any factors with insufficient component saturation. Prior to CFA, we eliminated one item (ADVICE) due to less than minimal variance. Subsequent *post hoc* model refinements resulted in the elimination of five additional items. Two items were eliminated for non-significant and non-salient factor loadings (SHOULDEV=0.075, p=0.513; COLLEFF=0.071, p=0.350), and three items were eliminated for non-salient factor loadings (OWNWELF=0.155, RESTRSER=-0.260, and DIFPROBS=-0.278). The standardised estimates of factor loadings from this model were acceptable (Table 3).

Additional details regarding the women's EFA and CFA results

During the EFA analyses, we eliminated one item (HAPPYNEI) due to less than minimal variance. We eliminated twelve additional items in a step-wise manner: six items were eliminated because they had no salient loadings on any factor (RESTRSER, BRIBELDR, COPARTCG, EXOASSIS, COMMGDEC, SHOULDEV); four items were complex variables that cross-loaded on more than one factor without sufficient substantive justification (SAFEATHO, SUPMOURN, SHAREKNO, TRUSTLDR); one item (PAREXOGP) was eliminated due to evidence of extreme multicollinearity with another item that loaded to the factor; and one item (ONWELF) was eliminated because although its pattern coefficient was salient, its structure coefficient was not salient on the factor of interest. This item reduction process resulted in a 37-item women's CE measurement model that tapped to seven factors of CE: social networks & reciprocity, social disorder, social attachment & personal agency, social response, associational participation, common vision, and community organisation & leadership.

Factor one, labelled "social networks & reciprocity" corresponded to the cognitive social capital domain, though it also tapped to certain aspects of structural social capital, as it reflected perceptions related to collections of individuals that promote and protect mutual or personal interests. The factor contained eight items that indicate various aspects of reciprocity demonstrated through social networks, the strength of personal relationships, and the community's propensity to contribute to community development.

Factors two and four corresponded to the informal social control domain, though factor four also tapped to certain aspects of cognitive social capital. Factor two, labelled "social disorder" contained three items that reflect the level of disorder in the community, including conflicts such as stealing, fighting, cheating, and problems caused by intolerance of differences amongst people. Factor four, labelled "social response" contained eight items that tap to various facets of perceptions regarding the community's propensity to address internal issues, including willingness to intervene when crime-like activities are observed, conflict-resolution, common moral principles and codes of behaviour, support in times of crisis, community trust, and strength of relationships.

Factors three and six corresponded to the social cohesion domain. Factor three, labelled "social attachment & personal agency" included six items that tap to concepts related to place identity, community acceptance and attachment, and personal agency. This suggests that, for women, one's sense of self-agency is linked to one's sense of belonging or social attachment. Factor six, labelled "common vision" is comprised of five items that reflect shared culture (common values, hopes for the future, ideas about how the community should be managed), social equity (equal distribution of goods in times of crisis), and perceptions regarding community-level agency.

Factors five and seven corresponded to the structural social capital domain. Factor five, labelled as "associational participation" related to the respondent's personal involvement in established

community structures – both exogenously and endogenously organised. The three constituent items reflect both membership (as indicated by meeting attendance) and participation in associational activities. Factor seven, labelled "community organisation & leadership" corresponded to various aspects of organisation within the community, including the activity level of endogenously organised community associations and leaders thereof, and community-selected representation.

We conducted CFA on the 37 items tapping to seven factors, as indicated by the EFA-derived women's CE factor solution. We moved forward with *post hoc* model refinements to eliminate non-salient and non-significant factor loadings as well as any factors with insufficient component saturation. This resulted in the elimination of five items and one factor. One item was eliminated for a non-significant and non-salient factor loading (CLOSE=0.167, p=0.075), and two items were eliminated for non-salient factor loadings (CHEATS=0.213, and SIMBLIEF=0.309). With the elimination of one non-saliently loading item to the social order factor, the factor itself failed to demonstrate sufficient component saturation, so the factor and its remaining two items (DIFPROBS=0.900, p=0.001 and CRIMECON=0.366, p=0.001) were eliminated from the women's measurement model. The standardised estimates of factor loadings for the resulting six-factor model were acceptable (Table 4). Modification Indices above 3.84 on the women's model were all relatively low, meaning localised strain was relatively low in all areas identified. No further modifications were made.

Additional details regarding comparison of men's and women's CE measurement models

The men's CE measurement model included one more factor (community leadership) than was indicated by the women's CE measurement model. Two of the three items that comprised the leadership factor in the men's model are included in the community organisation factor in the women's measurement model, as there was sufficient substantive justification for those items tapping to that factor.

Comparison of CFA results of our hypothesised CE framework vs. EFA-derived factor solutions

Fit statistics from the preliminary CFA of our hypothesised CE framework and the CFA of the EFA-derived factor solution suggest that slight revisions that were substantively justified resulted in valid CE measurement models for both men and women in the Ethiopian context (Appendix D).

Comparison of fit statistics for CFA of refined, single-group and parsimonious models

Given it is encouraged to consider numerous alternatives before settling on final measurement models [6], we performed a CFA on both men's and women's models that reflected the more parsimonious set of CE indicators (i.e., only those that were completely overlapping between refined and validated CFA models). We present model fit statistics for those models, and compare them to the refined, validated CFA models in Appendix E. These results indicate that both the more saturated and parsimonious models are valid CE measurement metrics. The gender-specific saturated models represent slightly better fitting models.

We present model fit statistics, unstandardised B, standard errors, and standardised β for competing MIMIC models in Appendix F.

ADDITIONAL DISCUSSION

Establishing this CE measurement scale in the early phases of the *Andilaye* trial allowed us to measure and assess collective efficacy at baseline, prior to the implementation of a community-level demand-side sanitation and hygiene intervention. We plan to employ this validated scale again at endline, and compare changes in CE measures between intervention and counterfactual communities over time (pre-, post-intervention). This will allow us to test our hypothesis that there is a bi-directional, causal association between CE and intervention effectiveness.

Further discussion of gender-specific CE measurement models

There were slight differences between gender-specific CE measurement models (31-item, seven-factor solution vs. 33-item, six factor solution for men and women). Major differences between men's and women's CE measurement models involved: 1) the number of factors included in the measurement model, and 2) the manner in which individual-level behavioural control items (SELFEFF, SEDEV) correlated with factors related to social networks versus social attachment for men and women, respectively. The ordering of the CE scale factors also differed between men's and women's measurement models, and social networks & reciprocity emerging as the first factor in the women's model while social response emerging as the first factor in the men's model. These types of differences were expected, and are supported by empirical evidence that suggests women have a higher dependence on social networks and "the commons" than men [14].

The women's CE measurement model included several additional items that tapped to its social network factor that were not included in the men's measurement model. These items reflected additional concepts that reflected facets of reciprocity, communal contribution and collaboration, and solidarity. The women's measurement model also indicated that willingness to intervene in situations of delinquent behaviour was an important item related to social response, and perceptions regarding a sense of pride in being part of one's community was an important item related to social attachment. These items were not indicated in the men's measurement model, though at least in the case of the item that corresponded to pride, the exclusion of that indicator may have to do with less than minimal variance amongst the item responses, as one response category for each of the split-half samples had no observations. The men's measurement model included two items that tapped to its social response factor that were not included in the women's measurement model. These items reflected common understanding regarding right and wrong and information sharing.

The men's measurement model also indicated that perceptions regarding normative expectations about members of the community working together to develop the community was an important item related to the common vision factor. Men's and women's measurement models differed in the sense that the men's CE measurement model indicated that a seventh factor – community leadership – was important for measuring CE. Two of the items that were included in this factor – those indicating supportive formal leadership and strong informal leadership – were included elsewhere in the women's measurement model (community organisation, as supported by sufficient substantive justification). A third item related to perceived trust in the community's leaders was not included in the women's measurement model, but was indicated as an important component for the measurement of community leadership in the men's model.

While we did reveal the underlying CE factor structure for gender-specific models, we also determined that there was considerable overlap between men's and women's CE measurement models. We determined that a parsimonious model that reflected all factors and items in common between the two gender-specific models demonstrated good model fit, and may therefore be used to measure and compare CE between genders. That said, the use of gender-specific CE scales may allow interested researchers to assess the mechanisms through which CE operate, and monitor how measures related to these gender-specific mechanisms change over time, throughout the duration of a development programme or research study.

Significant differences in associational participation factor scores corroborate existing evidence that suggests women may participate less in endogenous and exogenous community structures. This findings indicates that working through formal community structures to enhance women's behavioural control perspectives, including self- and collective efficacy, may not be an appropriate approach. More appropriate approaches may include community-level or household-level intervention activities.

In terms of selecting a CE measurement metric for administration more broadly, it is necessary to determine the aim and objectives of the work at hand, and weigh the benefits of being able to compare CE scores across genders (refined parsimonious CE scale) against being able to assess the mechanisms through which CE operate (gender-specific, saturated CE scales). Our results indicated that CE perceptions differ between men and women, even amongst those living in the same household. Therefore, researchers and programme implementers using an adapted version of our

parsimonious CE scale should either consider obtaining data from men and women within the same household or obtaining CE data from a random selection of men and women within a given community.

Additional discussion regarding factor indeterminacy

While the refined and final validated factor structures championed by this study demonstrate good model fit, and are substantively justified, they reflect only one possible representation of the relationship amongst items in the men's, women's, and parsimonious CE measurement models. As with any EFA, our results were influenced by the structure of the data for the particular sample we ascertained. Other measurement models that fit the data and represent the conceptualisation of CE as well or better than our refined gender-specific and final parsimonious CE measurement models may exist [6]. Through the employment of a randomly selected split-half hold-out sample, we sought to assess the stability of our EFA-derived CE factor structures across an independent sample from the same population, as suggested by numerous methodologists [4, 7, 15].

Further discussion of analytical limitations

Mathematically-focused factor extraction methods have a tendency to under- or over-estimate the number of factors in a solution [2, 4, 8]. The results of scree tests are often ambiguous (e.g., no clear shift in the slope) and subject to interpretation [4]. As a result, we used a combination of mathematical (i.e., eigenvalue-based Kaiser-Guttman rule), heuristic (i.e., scree plot), statistical (i.e., model fit statistics), and substantive justification to guide factor extraction. That said, we were not able to perform more rigorous procedures (e.g., parallel analysis) to confirm that we extract the correct number of factors, as these analyses are not available for categorical data in Mplus [16].

Sufficient component saturation is needed (i.e., two or more items with salient factor loadings) to guarantee appropriate factor interpretation [7]. While some methodologist suggest that as few as two to three items provide sufficient component saturation [17], other more conservative guidelines suggest four or more items with factor loadings of 0.5 or higher, and an average factor loading of 0.700 across all items tapping a factor. All six factors in the final parsimonious CE measurement model had three to five items per factor, all loading ≥ 0.478, indicating sufficient component saturation. With the except of one factor (i.e., "social networks", average factor loading = 0.663), all factors demonstrated average factor loadings of 0.700, signaling that the items were good measures of the factors to which they tapped. All seven factors of the refined, validated men's CE measurement model and all six factors of the refined, validated women's measurement model included three or more items, all with factor loadings greater than 0.500. However, one factor on the women's model, and four factors on the men's model included three items only, which just satisfies moderate [17], but does not more conservative guidelines for component saturation. In addition, two items within the men's measurement model, and one item within the women measurement model reflect factor loadings falling within the salient but only "adequate" range (i.e., 0.400-0.440). More importantly, perhaps, two factors in the refined, validated men's measurement model, and one factor in the refined, validated women's measurement model demonstrated average factor loadings below the ideal 0.700 average (average factor loading on refined CFA: 0.668 and 0.634 on the men's model; 0.656 on the women's model). Interestingly, the factor on the women's model and one factor on the men's model with average factor loadings less than 0.700 represented the social response factor. This suggests that perhaps the items we included in our CE survey for this CE sub-construct may not have included one or more proximal indicators of social response in the Ethiopian context.

Given our EFA results reflect the structure of the sample we ascertained, and the role that sampling error and other systematic error may play in the estimation of factor analytic results, initial EFA findings should be interpreted with caution. These findings should be cross-validated through additional EFA or CFAs using independent datasets [4]. We employed a random split-half hold-out sample for measurement model validation, and the resulting findings were promising, especially our refined final parsimonious CE measurement scale. Still, these findings should undergo further validation with independent datasets, which is planned for another WASH study being evaluated by

members of our research group. Since our results indicated that only minimal component saturation was attained for some CE factors, and more proximal indicators may not have been included for social response and social network factors, additional formative work that further explores these issues is warranted.

SUPPLEMENTAL MATERIAL REFERENCES

1. Thurstone, L.L., *MUltiple-factor analysis*. 1947, Chicago; London: University of Chicago Press.
2. Gorsuch, R.L., *Factor analysis.* 2013.
3. Tabachnick, B.G. and L.S. Fidell, *Using multivariate statistics*. 2007, Boston: Pearson/Allyn & Bacon.
4. Brown, T.A., *Confirmatory Factor Analysis for Applied Research*. 2006: Guilford Publications.
5. Kaiser, H.F., *The Application of Electronic Computers to Factor Analysis.* Educational and Psychological Measurement, 1960. **20**(1): p. 141-151.
6. Hancock, G.R. and R.O. Mueller, *The reviewer's guide to quantitative methods in the social sciences*. 2010, New York: Routledge.
7. Henson, R.K. and J.K. Roberts, *Use of Exploratory Factor Analysis in Published Research:Common Errors and Some Comment on Improved Practice.* Educational and Psychological Measurement, 2006. **66**(3): p. 393-416.
8. Comrey, A.L. and H.B. Lee, *A first course in factor analysis*. Second Edition ed. 1992, Hillsdale, NJ: Lawrence Erlbaum.
9. Li, C.-H., *Confirmatory factor analysis with ordinal data: Comparing robust maximum likelihood and diagonally weighted least squares.* Behavior Research Methods, 2016. **48**(3): p. 936-949.
10. Bollen, K.A., *Structural Equations with Latent Variables*. 1989: Wiley & Sons Inc.
11. Steinmetz, H., *Estimation and comparison of latent means across cultures*, in *Cross-Cultural Analysis : Methods and Applications*, E. Davidov, P. Schmidt, and J. Billiet, Editors. 2011, Taylor & Francis Group: New York, NY. p. 85-116.
12. Steinmetz, H., *Personal communication*. 2018.
13. Forrest, R. and A. Kearns, *Social Cohesion, Social Capital and the Neighbourhood.* Urban Studies, 2001. **38**(12): p. 2125-2143.
14. Agarwal, B., *Conceptualising environmental collective action: why gender matters.* Cambridge Journal of Economics, 2000. **24**(3): p. 283-310.
15. Baglin, J., *Improving Your Exploratory Factor Analysis for Ordinal Data: A Demonstration Using FACTOR.* Practical Assessment, Research & Evaluation, 2014. **19**(5).
16. Muthen, L.K. and B. Muthen, *Mplus : statistical analysis with latent variables : user's guide*. 2015.
17. Pedhazur, E.J. and F.N. Kerlinger, *Multiple regression in behavioral research: explanation and prediction*. 1982: Holt, Rinehart, and Winston.

© 2018 by the authors. Submitted for possible open access publication under the terms and conditions of the Creative Commons Attribution (CC BY) license (http://creativecommons.org/licenses/by/4.0/).

International Journal of
Environmental Research and Public Health

MDPI

Table S1. Univariate descriptive statistics: Frequency of responses by split-halves and gender.

		N		EFA sub-sample N_1						CFA sub-sample N_2					
		**Total sample**		**Aggregate**		**Women**		**Men**		**Aggregate**		**Women**		**Men**	
**CE item**	**Item response**	**N = 1,831**		n_{E1} **= 921**		n_{EW1} **= 366**		n_{EM1} **= 555**		n_{E2} **= 910**		n_{EW2} **= 360**		n_{EM2} **= 550**	
HARMONY	***People in this community live in harmony with each other most of the time.***														
	Completely disagree	147	8.03%	66	7.17%	22	6.01%	44	7.93%	81	8.90%	26	7.22%	55	10.00%
	Mildly/partially disagree	76	4.15%	41	4.45%	13	3.55%	28	5.05%	35	3.85%	15	4.17%	20	3.64%
	Neither agree nor disagree	20	1.09%	6	0.65%	5	1.37%	1	0.18%	14	1.54%	10	2.78%	4	0.73%
	Mildly/partially agree	710	38.78%	379	41.15%	112	30.60%	267	48.11%	331	36.37%	82	22.78%	249	45.27%
	Completely agree	878	47.95%	429	46.58%	214	58.47%	215	38.74%	449	49.34%	227	63.06%	222	40.36%
CHEATS	***In this community, you have to be careful, otherwise your neighbours may cheat you.***														
	Completely disagree	491	26.82%	245	26.60%	109	29.78%	136	24.50%	246	27.03%	105	29.17%	141	25.64%
	Mildly/partially disagree	174	9.50%	80	8.69%	37	10.11%	43	7.75%	94	10.33%	42	11.67%	52	9.45%
	Neither agree nor disagree	31	1.69%	15	1.63%	13	3.55%	2	0.36%	16	1.76%	9	2.50%	7	1.27%
	Mildly/partially agree	467	25.51%	241	26.17%	87	23.77%	154	27.75%	226	24.84%	95	26.39%	131	23.82%
	Completely agree	668	36.48%	340	36.92%	120	32.79%	220	39.64%	328	36.04%	109	30.28%	219	39.82%
CRIMECON	***In this community, conflicts like stealing and fighting often occur.***														
	Completely disagree	662	36.16%	319	34.64%	165	45.08%	154	27.75%	343	37.69%	179	49.72%	164	29.82%
	Mildly/partially disagree	146	7.97%	73	7.93%	44	12.02%	29	5.23%	73	8.02%	41	11.39%	32	5.82%
	Neither agree nor disagree	25	1.37%	12	1.30%	11	3.01%	1	0.18%	13	1.43%	6	1.67%	7	1.27%
	Mildly/partially agree	599	32.71%	316	34.31%	77	21.04%	239	43.06%	283	31.10%	72	20.00%	211	38.36%
	Completely agree	399	21.79%	201	21.82%	69	18.85%	132	23.78%	198	21.76%	62	17.22%	136	24.73%
SAFEATHO	***When I am at home alone, I feel safe from threats of crime.***														
	Completely disagree	375	20.48%	197	21.39%	92	25.14%	105	18.92%	178	19.56%	80	22.22%	98	17.82%

	Mildly/partially disagree	113	6.17%	67	7.27%	38	10.38%	29	5.23%	46	5.05%	28	7.78%	18	3.27%
	Neither agree nor disagree	60	3.28%	23	2.50%	17	4.64%	6	1.08%	37	4.07%	30	8.33%	7	1.27%
	Mildly/partially agree	379	20.70%	194	21.06%	88	24.04%	106	19.10%	185	20.33%	82	22.78%	103	18.73%
	Completely agree	904	49.37%	440	47.77%	131	35.79%	309	55.68%	464	50.99%	140	38.89%	324	58.91%
SIMBLIEF	***Most people in this community have similar beliefs about what is right and what is wrong.***														
	Completely disagree	325	17.75%	175	19.00%	57	15.57%	118	21.26%	150	16.48%	47	13.06%	103	18.73%
	Mildly/partially disagree	195	10.65%	100	10.86%	28	7.65%	72	12.97%	95	10.44%	26	7.22%	69	12.55%
	Neither agree nor disagree	128	6.99%	56	6.08%	43	11.75%	13	2.34%	72	7.91%	56	15.56%	16	2.91%
	Mildly/partially agree	651	35.55%	336	36.48%	127	34.70%	209	37.66%	315	34.62%	106	29.44%	209	38.00%
	Completely agree	532	29.06%	254	27.58%	111	30.33%	143	25.77%	278	30.55%	125	34.72%	153	27.82%
INTERCRI	***If the people of this community see crime-like activities, they will do something about it.***														
	Completely disagree	186	10.16%	84	9.12%	31	8.47%	53	9.55%	102	11.21%	35	9.72%	67	12.18%
	Mildly/partially disagree	91	4.97%	52	5.65%	21	5.74%	31	5.59%	39	4.29%	19	5.28%	20	3.64%
	Neither agree nor disagree	83	4.53%	42	4.56%	32	8.74%	10	1.80%	41	4.51%	30	8.33%	11	2.00%
	Mildly/partially agree	563	30.75%	294	31.92%	119	32.51%	175	31.53%	269	29.56%	98	27.22%	171	31.09%
	Completely agree	908	49.59%	449	48.75%	163	44.54%	286	51.53%	459	50.44%	178	49.44%	281	51.09%
SLVDISPU	***If there is a big dispute between two persons, other people from the community will help in solving the problem.***														
	Completely disagree	73	3.99%	38	4.13%	18	4.92%	20	3.60%	35	3.85%	17	4.72%	18	3.27%
	Mildly/partially disagree	42	2.29%	24	2.61%	11	3.01%	13	2.34%	18	1.98%	9	2.50%	9	1.64%
	Neither agree nor disagree	30	1.64%	15	1.63%	14	3.83%	1	0.18%	15	1.65%	12	3.33%	3	0.55%
	Mildly/partially agree	451	24.63%	217	23.56%	86	23.50%	131	23.60%	234	25.71%	89	24.72%	145	26.36%
	Completely agree	1235	67.45%	627	68.08%	237	64.75%	390	70.27%	608	66.81%	233	64.72%	375	68.18%
HLPCRPDZ	***If there is a problem that affects the entire community, for instance, crop disease, people in this community will help each other.***														
	Completely disagree	199	10.87%	101	10.97%	53	14.48%	48	8.65%	98	10.77%	47	13.06%	51	9.27%
	Mildly/partially disagree	64	3.50%	32	3.47%	13	3.55%	19	3.42%	32	3.52%	16	4.44%	16	2.91%
	Neither agree nor disagree	64	3.50%	30	3.26%	22	6.01%	8	1.44%	34	3.74%	24	6.67%	10	1.82%

	Mildly/partially agree	567	30.97%	290	31.49%	108	29.51%	182	32.79%	277	30.44%	88	24.44%	189	34.36%
	Completely agree	937	51.17%	468	50.81%	170	46.45%	298	53.69%	469	51.54%	185	51.39%	284	51.64%
SUPMOURN*[1]	***If someone in this community had a death in their family, the community will come together to support them while they mourn.***														
	Completely disagree	6	0.33%	3	0.33%	2	0.55%	1	0.18%	3	0.33%	2	0.56%	1	0.18%
	Mildly/partially disagree	8	0.44%	6	0.65%	3	0.82%	3	0.54%	2	0.22%	1	0.28%	1	0.18%
	Neither agree nor disagree	12	0.66%	6	0.65%	5	1.37%	1	0.18%	6	0.66%	5	1.39%	1	0.18%
	Mildly/partially agree	88	4.81%	55	5.97%	31	8.47%	24	4.32%	33	3.63%	19	5.28%	14	2.55%
	Completely agree	1717	93.77%	851	92.40%	325	88.80%	526	94.77%	866	95.16%	333	92.50%	533	96.91%
COMPRSLV	***When there is a problem in this community, people come together to discuss how it should be solved.***														
	Completely disagree	83	4.53%	43	4.67%	17	4.64%	26	4.68%	40	4.40%	11	3.06%	29	5.27%
	Mildly/partially disagree	37	2.02%	18	1.95%	7	1.91%	11	1.98%	19	2.09%	9	2.50%	10	1.82%
	Neither agree nor disagree	42	2.29%	16	1.74%	13	3.55%	3	0.54%	26	2.86%	20	5.56%	6	1.09%
	Mildly/partially agree	555	30.31%	286	31.05%	99	27.05%	187	33.69%	269	29.56%	93	25.83%	176	32.00%
	Completely agree	1114	60.84%	558	60.59%	230	62.84%	328	59.10%	556	61.10%	227	63.06%	329	59.82%
CONTRDEV	***The people of this community will contribute their own money or labour for community development.***														
	Completely disagree	113	6.17%	55	5.97%	21	5.74%	34	6.13%	58	6.37%	16	4.44%	42	7.64%
	Mildly/partially disagree	61	3.33%	30	3.26%	19	5.19%	11	1.98%	31	3.41%	12	3.33%	19	3.45%
	Neither agree nor disagree	48	2.62%	26	2.82%	19	5.19%	7	1.26%	22	2.42%	20	5.56%	2	0.36%
	Mildly/partially agree	654	35.72%	349	37.89%	126	34.43%	223	40.18%	305	33.52%	116	32.22%	189	34.36%
	Completely agree	955	52.16%	461	50.05%	181	49.45%	280	50.45%	494	54.29%	196	54.44%	298	54.18%
DIFPROBS	***Differences between people, such as the amount of land they own, often causes problems in this community.***														
	Completely disagree	372	20.32%	195	21.17%	72	19.67%	123	22.16%	177	19.45%	72	20.00%	105	19.09%
	Mildly/partially disagree	99	5.41%	50	5.43%	32	8.74%	18	3.24%	49	5.38%	28	7.78%	21	3.82%
	Neither agree nor disagree	74	4.04%	41	4.45%	37	10.11%	4	0.72%	33	3.63%	23	6.39%	10	1.82%
	Mildly/partially agree	479	26.16%	256	27.80%	89	24.32%	167	30.09%	223	24.51%	78	21.67%	145	26.36%
	Completely agree	807	44.07%	379	41.15%	136	37.16%	243	43.78%	428	47.03%	159	44.17%	269	48.91%

HAPPYNEI *[1]	***I feel happy for my neighbour if they have a good harvest.***														
	Completely disagree	13	0.71%	8	0.87%	8	2.19%	0	0.00%	5	0.55%	4	1.11%	1	0.18%
	Mildly/partially disagree	4	0.22%	2	0.22%	1	0.27%	1	0.18%	2	0.22%	0	0.00%	2	0.36%
	Neither agree nor disagree	12	0.66%	7	0.76%	5	1.37%	2	0.36%	5	0.55%	5	1.39%	0	0.00%
	Mildly/partially agree	78	4.26%	46	4.99%	24	6.56%	22	3.96%	32	3.52%	13	3.61%	19	3.45%
	Completely agree	1724	94.16%	858	93.16%	328	89.62%	530	95.50%	866	95.16%	338	93.89%	528	96.00%
COMTRUST	***People in this community can be trusted.***														
	Completely disagree	90	4.92%	42	4.56%	18	4.92%	24	4.32%	48	5.27%	23	6.39%	25	4.55%
	Mildly/partially disagree	71	3.88%	42	4.56%	19	5.19%	23	4.14%	29	3.19%	14	3.89%	15	2.73%
	Neither agree nor disagree	58	3.17%	20	2.17%	12	3.28%	8	1.44%	38	4.18%	30	8.33%	8	1.45%
	Mildly/partially agree	772	42.16%	396	43.00%	127	34.70%	269	48.47%	376	41.32%	115	31.94%	261	47.45%
	Completely agree	840	45.88%	421	45.71%	190	51.91%	231	41.62%	419	46.04%	178	49.44%	241	43.82%
ADVICE	***I typically accept advice from others in this community.***														
	Completely disagree	18	0.98%	10	1.09%	8	2.19%	2	0.36%	8	0.88%	4	1.11%	4	0.73%
	Mildly/partially disagree	20	1.09%	7	0.76%	2	0.55%	5	0.90%	13	1.43%	11	3.06%	2	0.36%
	Neither agree nor disagree	17	0.93%	7	0.76%	6	1.64%	1	0.18%	10	1.10%	10	2.78%	0	0.00%
	Mildly/partially agree	646	35.28%	342	37.13%	149	40.71%	193	34.77%	304	33.41%	120	33.33%	184	33.45%
	Completely agree	1130	61.71%	555	60.26%	201	54.92%	354	63.78%	575	63.19%	215	59.72%	360	65.45%
SHAREKNO	***People in the community share new knowledge with their neighbour if they learn something new.***														
	Completely disagree	155	8.47%	65	7.06%	19	5.19%	46	8.29%	90	9.89%	35	9.72%	55	10.00%
	Mildly/partially disagree	77	4.21%	32	3.47%	10	2.73%	22	3.96%	45	4.95%	26	7.22%	19	3.45%
	Neither agree nor disagree	82	4.48%	37	4.02%	16	4.37%	21	3.78%	45	4.95%	28	7.78%	17	3.09%
	Mildly/partially agree	630	34.41%	335	36.37%	135	36.89%	200	36.04%	295	32.42%	103	28.61%	192	34.91%
	Completely agree	887	48.44%	452	49.08%	186	50.82%	266	47.93%	435	47.80%	168	46.67%	267	48.55%
CLOSE	***This is a close-knit community (i.e., people in this community have close personal relationships with each other).***														
	Completely disagree	61	3.33%	32	3.47%	17	4.64%	15	2.70%	29	3.19%	10	2.78%	19	3.45%

	Mildly/partially disagree	60	3.28%	32	3.47%	13	3.55%	19	3.42%	28	3.08%	14	3.89%	14	2.55%
	Neither agree nor disagree	36	1.97%	15	1.63%	12	3.28%	3	0.54%	21	2.31%	17	4.72%	4	0.73%
	Mildly/partially agree	684	37.36%	352	38.22%	126	34.43%	226	40.72%	332	36.48%	124	34.44%	208	37.82%
	Completely agree	990	54.07%	490	53.20%	198	54.10%	292	52.61%	500	54.95%	195	54.17%	305	55.45%
OWNWELF	***In this community, people prioritise their own family's welfare over community development.***														
	Completely disagree	119	6.50%	56	6.08%	25	6.83%	31	5.59%	63	6.92%	26	7.22%	37	6.73%
	Mildly/partially disagree	60	3.28%	40	4.34%	27	7.38%	13	2.34%	20	2.20%	11	3.06%	9	1.64%
	Neither agree nor disagree	54	2.95%	26	2.82%	24	6.56%	2	0.36%	28	3.08%	20	5.56%	8	1.45%
	Mildly/partially agree	392	21.41%	207	22.48%	108	29.51%	99	17.84%	185	20.33%	96	26.67%	89	16.18%
	Completely agree	1206	65.87%	592	64.28%	182	49.73%	410	73.87%	614	67.47%	207	57.50%	407	74.00%
LOSTCOW *[1]	***If someone in this community loses a cow or goat, a neighbour will help look for it.***														
	Completely disagree	10	0.55%	5	0.54%	2	0.55%	3	0.54%	5	0.55%	5	1.39%	0	0.00%
	Mildly/partially disagree	6	0.33%	4	0.43%	2	0.55%	2	0.36%	2	0.22%	1	0.28%	1	0.18%
	Neither agree nor disagree	10	0.55%	5	0.54%	4	1.09%	1	0.18%	5	0.55%	5	1.39%	0	0.00%
	Mildly/partially agree	172	9.39%	80	8.69%	26	7.10%	54	9.73%	92	10.11%	26	7.22%	66	12.00%
	Completely agree	1633	89.19%	827	89.79%	332	90.71%	495	89.19%	806	88.57%	323	89.72%	483	87.82%
BORMONEY	***If you suddenly need some money, you can borrow from a person or group in your community.***														
	Completely disagree	153	8.36%	78	8.47%	36	9.84%	42	7.57%	75	8.24%	33	9.17%	42	7.64%
	Mildly/partially disagree	47	2.57%	18	1.95%	9	2.46%	9	1.62%	29	3.19%	16	4.44%	13	2.36%
	Neither agree nor disagree	22	1.20%	11	1.19%	5	1.37%	6	1.08%	11	1.21%	8	2.22%	3	0.55%
	Mildly/partially agree	481	26.27%	243	26.38%	93	25.41%	150	27.03%	238	26.15%	83	23.06%	155	28.18%
	Completely agree	1128	61.61%	571	62.00%	223	60.93%	348	62.70%	557	61.21%	220	61.11%	337	61.27%
NEICAREG	***If you and your relatives suddenly had to go away for a day or two, you could count on your neighbours to take care of your children.***														
	Completely disagree	109	5.95%	48	5.21%	22	6.01%	26	4.68%	61	6.70%	31	8.61%	30	5.45%
	Mildly/partially disagree	42	2.29%	22	2.39%	6	1.64%	16	2.88%	20	2.20%	8	2.22%	12	2.18%
	Neither agree nor disagree	26	1.42%	11	1.19%	4	1.09%	7	1.26%	15	1.65%	5	1.39%	10	1.82%

	Mildly/partially agree	394	21.52%	198	21.50%	80	21.86%	118	21.26%	196	21.54%	75	20.83%	121	22.00%
	Completely agree	1260	68.81%	642	69.71%	254	69.40%	388	69.91%	618	67.91%	241	66.94%	377	68.55%
UNOFLDRS	***There are people in this community who show strong leadership.***														
	Completely disagree	64	3.50%	27	2.93%	9	2.46%	18	3.24%	37	4.07%	15	4.17%	22	4.00%
	Mildly/partially disagree	50	2.73%	23	2.50%	10	2.73%	13	2.34%	27	2.97%	13	3.61%	14	2.55%
	Neither agree nor disagree	112	6.12%	44	4.78%	33	9.02%	11	1.98%	68	7.47%	50	13.89%	18	3.27%
	Mildly/partially agree	758	41.40%	407	44.19%	141	38.52%	266	47.93%	351	38.57%	118	32.78%	233	42.36%
	Completely agree	847	46.26%	420	45.60%	173	47.27%	247	44.50%	427	46.92%	164	45.56%	263	47.82%
COMACTCG*	***The community-based associations, such as the Edir, in this community is very active.***														
	Completely disagree	26	1.42%	10	1.09%	6	1.64%	4	0.72%	16	1.76%	5	1.39%	11	2.00%
	Mildly/partially disagree	19	1.04%	7	0.76%	3	0.82%	4	0.72%	12	1.32%	7	1.94%	5	0.91%
	Neither agree nor disagree	39	2.13%	24	2.61%	16	4.37%	8	1.44%	15	1.65%	13	3.61%	2	0.36%
	Mildly/partially agree	293	16.00%	145	15.74%	76	20.77%	69	12.43%	148	16.26%	88	24.44%	60	10.91%
	Completely agree	1454	79.41%	735	79.80%	265	72.40%	470	84.68%	719	79.01%	247	68.61%	472	85.82%
ACTLDR1	***The leaders of community-based associations, like Edir leaders, respond to this community's concerns.***														
	Completely disagree	38	2.08%	15	1.63%	5	1.37%	10	1.80%	23	2.53%	8	2.22%	15	2.73%
	Mildly/partially disagree	20	1.09%	10	1.09%	7	1.91%	3	0.54%	10	1.10%	5	1.39%	5	0.91%
	Neither agree nor disagree	76	4.15%	42	4.56%	28	7.65%	14	2.52%	34	3.74%	27	7.50%	7	1.27%
	Mildly/partially agree	408	22.28%	215	23.34%	98	26.78%	117	21.08%	193	21.21%	97	26.94%	96	17.45%
	Completely agree	1289	70.40%	639	69.38%	228	62.30%	411	74.05%	650	71.43%	223	61.94%	427	77.64%
ACTLDR2	***Formal administrative leaders, like the kebele manager, provide support to this community.***														
	Completely disagree	125	6.83%	59	6.41%	19	5.19%	40	7.21%	66	7.25%	25	6.94%	41	7.45%
	Mildly/partially disagree	71	3.88%	33	3.58%	14	3.83%	19	3.42%	38	4.18%	15	4.17%	23	4.18%
	Neither agree nor disagree	139	7.59%	64	6.95%	50	13.66%	14	2.52%	75	8.24%	57	15.83%	18	3.27%
	Mildly/partially agree	644	35.17%	332	36.05%	123	33.61%	209	37.66%	312	34.29%	108	30.00%	204	37.09%
	Completely agree	852	46.53%	433	47.01%	160	43.72%	273	49.19%	419	46.04%	155	43.06%	264	48.00%

TRUSTLDR	***This community's leaders can be trusted.***														
	Completely disagree	97	5.30%	45	4.89%	17	4.64%	28	5.05%	52	5.71%	15	4.17%	37	6.73%
	Mildly/partially disagree	73	3.99%	36	3.91%	16	4.37%	20	3.60%	37	4.07%	19	5.28%	18	3.27%
	Neither agree nor disagree	101	5.52%	40	4.34%	27	7.38%	13	2.34%	61	6.70%	48	13.33%	13	2.36%
	Mildly/partially agree	722	39.43%	377	40.93%	137	37.43%	240	43.24%	345	37.91%	110	30.56%	235	42.73%
	Completely agree	838	45.77%	423	45.93%	169	46.17%	254	45.77%	415	45.60%	168	46.67%	247	44.91%
CHOCGLDR*	***People in this community get to choose the leaders of their own community-based associations, such as the Edir leaders.***														
	Completely disagree	32	1.75%	12	1.30%	6	1.64%	6	1.08%	20	2.20%	6	1.67%	14	2.55%
	Mildly/partially disagree	12	0.66%	7	0.76%	6	1.64%	1	0.18%	5	0.55%	3	0.83%	2	0.36%
	Neither agree nor disagree	49	2.68%	22	2.39%	13	3.55%	9	1.62%	27	2.97%	20	5.56%	7	1.27%
	Mildly/partially agree	254	13.87%	137	14.88%	76	20.77%	61	10.99%	117	12.86%	66	18.33%	51	9.27%
	Completely agree	1484	81.05%	743	80.67%	265	72.40%	478	86.13%	741	81.43%	265	73.61%	476	86.55%
HAVEFRND	***In this community, I have friends with whom I can share my problems.***														
	Completely disagree	90	4.92%	41	4.45%	25	6.83%	16	2.88%	49	5.38%	29	8.06%	20	3.64%
	Mildly/partially disagree	22	1.20%	12	1.30%	8	2.19%	4	0.72%	10	1.10%	6	1.67%	4	0.73%
	Neither agree nor disagree	14	0.76%	8	0.87%	8	2.19%	0	0.00%	6	0.66%	6	1.67%	0	0.00%
	Mildly/partially agree	464	25.34%	239	25.95%	95	25.96%	144	25.95%	225	24.73%	87	24.17%	138	25.09%
	Completely agree	1241	67.78%	621	67.43%	230	62.84%	391	70.45%	620	68.13%	232	64.44%	388	70.55%
COME4HLP	***My neighbours sometimes come to me to share their problems and get help.***														
	Completely disagree	88	4.81%	42	4.56%	23	6.28%	19	3.42%	46	5.05%	26	7.22%	20	3.64%
	Mildly/partially disagree	32	1.75%	15	1.63%	9	2.46%	6	1.08%	17	1.87%	11	3.06%	6	1.09%
	Neither agree nor disagree	15	0.82%	6	0.65%	5	1.37%	1	0.18%	9	0.99%	8	2.22%	1	0.18%
	Mildly/partially agree	575	31.40%	284	30.84%	125	34.15%	159	28.65%	291	31.98%	118	32.78%	173	31.45%
	Completely agree	1121	61.22%	574	62.32%	204	55.74%	370	66.67%	547	60.11%	197	54.72%	350	63.64%
COPARTCG	***Most people in this community participate in community associations.***														
	Completely disagree	62	3.39%	35	3.80%	21	5.74%	14	2.52%	27	2.97%	16	4.44%	11	2.00%

	Mildly/partially disagree	35	1.91%	17	1.85%	10	2.73%	7	1.26%	18	1.98%	10	2.78%	8	1.45%
	Neither agree nor disagree	66	3.60%	26	2.82%	20	5.46%	6	1.08%	40	4.40%	29	8.06%	11	2.00%
	Mildly/partially agree	575	31.40%	297	32.25%	121	33.06%	176	31.71%	278	30.55%	104	28.89%	174	31.64%
	Completely agree	1093	59.69%	546	59.28%	194	53.01%	352	63.42%	547	60.11%	201	55.83%	346	62.91%
ACTCBGP	***I attend meetings of a community-based association, such as the Edir.***														
	Completely disagree	263	14.36%	126	13.68%	86	23.50%	40	7.21%	137	15.05%	92	25.56%	45	8.18%
	Mildly/partially disagree	44	2.40%	22	2.39%	15	4.10%	7	1.26%	22	2.42%	20	5.56%	2	0.36%
	Neither agree nor disagree	33	1.80%	16	1.74%	10	2.73%	6	1.08%	17	1.87%	13	3.61%	4	0.73%
	Mildly/partially agree	403	22.01%	205	22.26%	111	30.33%	94	16.94%	198	21.76%	101	28.06%	97	17.64%
	Completely agree	1088	59.42%	552	59.93%	144	39.34%	408	73.51%	536	58.90%	134	37.22%	402	73.09%
PARTCBGP	***I participate in activities held by any community-based associations, such as the Edir.***														
	Completely disagree	204	11.14%	92	9.99%	60	16.39%	32	5.77%	112	12.31%	66	18.33%	46	8.36%
	Mildly/partially disagree	49	2.68%	24	2.61%	14	3.83%	10	1.80%	25	2.75%	19	5.28%	6	1.09%
	Neither agree nor disagree	34	1.86%	15	1.63%	9	2.46%	6	1.08%	19	2.09%	17	4.72%	2	0.36%
	Mildly/partially agree	390	21.30%	206	22.37%	105	28.69%	101	18.20%	184	20.22%	96	26.67%	88	16.00%
	Completely agree	1154	63.03%	584	63.41%	178	48.63%	406	73.15%	570	62.64%	162	45.00%	408	74.18%
ACTEXOGP	***I attend the meetings of any government or NGO-initiated community development group, such as the Development Army.***														
	Completely disagree	315	17.20%	156	16.94%	104	28.42%	52	9.37%	159	17.47%	98	27.22%	61	11.09%
	Mildly/partially disagree	99	5.41%	46	4.99%	28	7.65%	18	3.24%	53	5.82%	32	8.89%	21	3.82%
	Neither agree nor disagree	53	2.89%	24	2.61%	16	4.37%	8	1.44%	29	3.19%	26	7.22%	3	0.55%
	Mildly/partially agree	437	23.87%	235	25.52%	104	28.42%	131	23.60%	202	22.20%	93	25.83%	109	19.82%
	Completely agree	927	50.63%	460	49.95%	114	31.15%	346	62.34%	467	51.32%	111	30.83%	356	64.73%
PAREXOGP	***I participate in activities held by any government or NGO-initiated community development group, such as the Development Army.***														
	Completely disagree	342	18.68%	169	18.35%	104	28.42%	65	11.71%	173	19.01%	108	30.00%	65	11.82%
	Mildly/partially disagree	106	5.79%	50	5.43%	30	8.20%	20	3.60%	56	6.15%	33	9.17%	23	4.18%
	Neither agree nor disagree	55	3.00%	25	2.71%	17	4.64%	8	1.44%	30	3.30%	27	7.50%	3	0.55%

	Mildly/partially agree	461	25.18%	240	26.06%	100	27.32%	140	25.23%	221	24.29%	96	26.67%	125	22.73%
	Completely agree	867	47.35%	437	47.45%	115	31.42%	322	58.02%	430	47.25%	96	26.67%	334	60.73%
COMMGDEC	***When community groups make decisions, they are pleasing and good for most of the households in this community.***														
	Completely disagree	87	4.75%	42	4.56%	24	6.56%	18	3.24%	45	4.95%	22	6.11%	23	4.18%
	Mildly/partially disagree	62	3.39%	31	3.37%	17	4.64%	14	2.52%	31	3.41%	19	5.28%	12	2.18%
	Neither agree nor disagree	131	7.15%	64	6.95%	42	11.48%	22	3.96%	67	7.36%	47	13.06%	20	3.64%
	Mildly/partially agree	707	38.61%	363	39.41%	145	39.62%	218	39.28%	344	37.80%	127	35.28%	217	39.45%
	Completely agree	844	46.10%	421	45.71%	138	37.70%	283	50.99%	423	46.48%	145	40.28%	278	50.55%
BRIBELDR	***Sometimes people need to bribe community leaders in order to get things done.***														
	Completely disagree	1163	63.52%	584	63.41%	187	51.09%	397	71.53%	579	63.63%	193	53.61%	386	70.18%
	Mildly/partially disagree	104	5.68%	61	6.62%	31	8.47%	30	5.41%	43	4.73%	21	5.83%	22	4.00%
	Neither agree nor disagree	207	11.31%	93	10.10%	65	17.76%	28	5.05%	114	12.53%	72	20.00%	42	7.64%
	Mildly/partially agree	185	10.10%	97	10.53%	37	10.11%	60	10.81%	88	9.67%	33	9.17%	55	10.00%
	Completely agree	172	9.39%	86	9.34%	46	12.57%	40	7.21%	86	9.45%	41	11.39%	45	8.18%
DISTCRIS	***During a crisis situation, such as a drought, government services are distributed equally by the community to all households in need.***														
	Completely disagree	185	10.10%	92	9.99%	29	7.92%	63	11.35%	93	10.22%	39	10.83%	54	9.82%
	Mildly/partially disagree	108	5.90%	57	6.19%	23	6.28%	34	6.13%	51	5.60%	17	4.72%	34	6.18%
	Neither agree nor disagree	292	15.95%	147	15.96%	74	20.22%	73	13.15%	145	15.93%	79	21.94%	66	12.00%
	Mildly/partially agree	553	30.20%	273	29.64%	104	28.42%	169	30.45%	280	30.77%	98	27.22%	182	33.09%
	Completely agree	693	37.85%	352	38.22%	136	37.16%	216	38.92%	341	37.47%	127	35.28%	214	38.91%
RESTRSER	***Some households in this community are restricted from community services, such as bed net distribution.***														
	Completely disagree	993	54.23%	496	53.85%	162	44.26%	334	60.18%	497	54.62%	173	48.06%	324	58.91%
	Mildly/partially disagree	192	10.49%	92	9.99%	42	11.48%	50	9.01%	100	10.99%	44	12.22%	56	10.18%
	Neither agree nor disagree	135	7.37%	64	6.95%	37	10.11%	27	4.86%	71	7.80%	48	13.33%	23	4.18%
	Mildly/partially agree	256	13.98%	133	14.44%	59	16.12%	74	13.33%	123	13.52%	53	14.72%	70	12.73%
	Completely agree	255	13.93%	136	14.77%	66	18.03%	70	12.61%	119	13.08%	42	11.67%	77	14.00%

COMMVALU	***Most people in this community have common values, for example, they value hard work.***														
	Completely disagree	36	1.97%	15	1.63%	6	1.64%	9	1.62%	21	2.31%	9	2.50%	12	2.18%
	Mildly/partially disagree	33	1.80%	15	1.63%	7	1.91%	8	1.44%	18	1.98%	11	3.06%	7	1.27%
	Neither agree nor disagree	75	4.10%	43	4.67%	33	9.02%	10	1.80%	32	3.52%	29	8.06%	3	0.55%
	Mildly/partially agree	573	31.29%	287	31.16%	126	34.43%	161	29.01%	286	31.43%	111	30.83%	175	31.82%
	Completely agree	1114	60.84%	561	60.91%	194	53.01%	367	66.13%	553	60.77%	200	55.56%	353	64.18%
SIMHOPES	***Most people in this community have similar hopes about the future development of the community.***														
	Completely disagree	56	3.06%	27	2.93%	12	3.28%	15	2.70%	29	3.19%	10	2.78%	19	3.45%
	Mildly/partially disagree	46	2.51%	27	2.93%	13	3.55%	14	2.52%	19	2.09%	13	3.61%	6	1.09%
	Neither agree nor disagree	132	7.21%	67	7.27%	48	13.11%	19	3.42%	65	7.14%	48	13.33%	17	3.09%
	Mildly/partially agree	637	34.79%	328	35.61%	133	36.34%	195	35.14%	309	33.96%	127	35.28%	182	33.09%
	Completely agree	960	52.43%	472	51.25%	160	43.72%	312	56.22%	488	53.63%	162	45.00%	326	59.27%
COMMGMT	***People in this community share the same ideas on how village matters should be managed.***														
	Completely disagree	63	3.44%	27	2.93%	13	3.55%	14	2.52%	36	3.96%	18	5.00%	18	3.27%
	Mildly/partially disagree	54	2.95%	25	2.71%	11	3.01%	14	2.52%	29	3.19%	16	4.44%	13	2.36%
	Neither agree nor disagree	117	6.39%	62	6.73%	45	12.30%	17	3.06%	55	6.04%	39	10.83%	16	2.91%
	Mildly/partially agree	635	34.68%	314	34.09%	126	34.43%	188	33.87%	321	35.27%	126	35.00%	195	35.45%
	Completely agree	962	52.54%	493	53.53%	171	46.72%	322	58.02%	469	51.54%	161	44.72%	308	56.00%
ACCEPT*	***People in this community accept me as a member of the community.***														
	Completely disagree	39	2.13%	17	1.85%	15	4.10%	2	0.36%	22	2.42%	16	4.44%	6	1.09%
	Mildly/partially disagree	27	1.47%	13	1.41%	6	1.64%	7	1.26%	14	1.54%	8	2.22%	6	1.09%
	Neither agree nor disagree	39	2.13%	20	2.17%	16	4.37%	4	0.72%	19	2.09%	17	4.72%	2	0.36%
	Mildly/partially agree	314	17.15%	164	17.81%	97	26.50%	67	12.07%	150	16.48%	85	23.61%	65	11.82%
	Completely agree	1412	77.12%	707	76.76%	232	63.39%	475	85.59%	705	77.47%	234	65.00%	471	85.64%
ATTACH*	***I feel attached to this community and its people.***														
	Completely disagree	31	1.69%	16	1.74%	10	2.73%	6	1.08%	15	1.65%	11	3.06%	4	0.73%

	Mildly/partially disagree	25	1.37%	7	0.76%	4	1.09%	3	0.54%	18	1.98%	9	2.50%	9	1.64%
	Neither agree nor disagree	25	1.37%	11	1.19%	9	2.46%	2	0.36%	14	1.54%	12	3.33%	2	0.36%
	Mildly/partially agree	327	17.86%	166	18.02%	100	27.32%	66	11.89%	161	17.69%	91	25.28%	70	12.73%
	Completely agree	1423	77.72%	721	78.28%	243	66.39%	478	86.13%	702	77.14%	237	65.83%	465	84.55%
PROUD*	***I feel proud to be part of this community.***														
	Completely disagree	40	2.18%	16	1.74%	12	3.28%	4	0.72%	24	2.64%	18	5.00%	6	1.09%
	Mildly/partially disagree	25	1.37%	8	0.87%	8	2.19%	0	0.00%	17	1.87%	10	2.78%	7	1.27%
	Neither agree nor disagree	18	0.98%	8	0.87%	5	1.37%	3	0.54%	10	1.10%	10	2.78%	0	0.00%
	Mildly/partially agree	219	11.96%	112	12.16%	70	19.13%	42	7.57%	107	11.76%	67	18.61%	40	7.27%
	Completely agree	1529	83.51%	777	84.36%	271	74.04%	506	91.17%	752	82.64%	255	70.83%	497	90.36%
IDENTITY*	***Being a member of this community is part of who I am.***														
	Completely disagree	27	1.47%	13	1.41%	9	2.46%	4	0.72%	14	1.54%	12	3.33%	2	0.36%
	Mildly/partially disagree	25	1.37%	9	0.98%	6	1.64%	3	0.54%	16	1.76%	10	2.78%	6	1.09%
	Neither agree nor disagree	35	1.91%	19	2.06%	15	4.10%	4	0.72%	16	1.76%	14	3.89%	2	0.36%
	Mildly/partially agree	211	11.52%	107	11.62%	67	18.31%	40	7.21%	104	11.43%	68	18.89%	36	6.55%
	Completely agree	1533	83.72%	773	83.93%	269	73.50%	504	90.81%	760	83.52%	256	71.11%	504	91.64%
SELFEFF*	***I have the capacity to achieve my future aims.***														
	Completely disagree	36	1.97%	19	2.06%	7	1.91%	12	2.16%	17	1.87%	9	2.50%	8	1.45%
	Mildly/partially disagree	13	0.71%	5	0.54%	4	1.09%	1	0.18%	8	0.88%	3	0.83%	5	0.91%
	Neither agree nor disagree	38	2.08%	20	2.17%	14	3.83%	6	1.08%	18	1.98%	11	3.06%	7	1.27%
	Mildly/partially agree	375	20.48%	187	20.30%	96	26.23%	91	16.40%	188	20.66%	86	23.89%	102	18.55%
	Completely agree	1369	74.77%	690	74.92%	245	66.94%	445	80.18%	679	74.62%	251	69.72%	428	77.82%
SEDEV	***I have the ability to contribute to this community's development.***														
	Completely disagree	66	3.60%	38	4.13%	22	6.01%	16	2.88%	28	3.08%	15	4.17%	13	2.36%
	Mildly/partially disagree	66	3.60%	25	2.71%	17	4.64%	8	1.44%	41	4.51%	30	8.33%	11	2.00%
	Neither agree nor disagree	92	5.02%	44	4.78%	35	9.56%	9	1.62%	48	5.27%	30	8.33%	18	3.27%

	Mildly/partially agree	544	29.71%	273	29.64%	111	30.33%	162	29.19%	271	29.78%	102	28.33%	169	30.73%
	Completely agree	1063	58.06%	541	58.74%	181	49.45%	360	64.86%	522	57.36%	183	50.83%	339	61.64%
COLLEFF	***People in this community have the capacity to make positive changes by coming together.***														
	Completely disagree	51	2.79%	21	2.28%	10	2.73%	11	1.98%	30	3.30%	11	3.06%	19	3.45%
	Mildly/partially disagree	34	1.86%	13	1.41%	5	1.37%	8	1.44%	21	2.31%	9	2.50%	12	2.18%
	Neither agree nor disagree	119	6.50%	63	6.84%	50	13.66%	13	2.34%	56	6.15%	44	12.22%	12	2.18%
	Mildly/partially agree	545	29.77%	276	29.97%	108	29.51%	168	30.27%	269	29.56%	114	31.67%	155	28.18%
	Completely agree	1082	59.09%	548	59.50%	193	52.73%	355	63.96%	534	58.68%	182	50.56%	352	64.00%
EXOASSIS	***This community needs assistance from others outside the community in order to make positive changes.***														
	Completely disagree	296	16.17%	144	15.64%	31	8.47%	113	20.36%	152	16.70%	45	12.50%	107	19.45%
	Mildly/partially disagree	84	4.59%	48	5.21%	27	7.38%	21	3.78%	36	3.96%	17	4.72%	19	3.45%
	Neither agree nor disagree	105	5.73%	57	6.19%	41	11.20%	16	2.88%	48	5.27%	38	10.56%	10	1.82%
	Mildly/partially agree	507	27.69%	251	27.25%	98	26.78%	153	27.57%	256	28.13%	108	30.00%	148	26.91%
	Completely agree	839	45.82%	421	45.71%	169	46.17%	252	45.41%	418	45.93%	152	42.22%	266	48.36%
SHOULDEV*	***People in this community should work together to develop the community.***														
	Completely disagree	36	1.97%	16	1.74%	6	1.64%	10	1.80%	20	2.20%	9	2.50%	11	2.00%
	Mildly/partially disagree	23	1.26%	9	0.98%	4	1.09%	5	0.90%	14	1.54%	7	1.94%	7	1.27%
	Neither agree nor disagree	44	2.40%	23	2.50%	19	5.19%	4	0.72%	21	2.31%	17	4.72%	4	0.73%
	Mildly/partially agree	319	17.42%	161	17.48%	67	18.31%	94	16.94%	158	17.36%	71	19.72%	87	15.82%
	Completely agree	1409	76.95%	712	77.31%	270	73.77%	442	79.64%	697	76.59%	256	71.11%	441	80.18%

Notes. * Items with skewness outside the suggested range (i.e., > 3.0), distributions of aggregate men's CE item responses

ı Items with skewness outside the suggested range (i.e., > 3.0), distributions of aggregate women's CE items responses

2
